# Socializing AI: Integrating Social Network Analysis and Deep Learning for Precision Dairy Cow Monitoring—A Critical Review

**DOI:** 10.3390/ani15131835

**Published:** 2025-06-20

**Authors:** Sibi Chakravathy Parivendan, Kashfia Sailunaz, Suresh Neethirajan

**Affiliations:** 1 Faculty of Computer Science, Dalhousie University, 6050 University Avenue, Halifax, NS B3H 4R2, Canada; 2 Faculty of Agriculture, Dalhousie University, Truro, NS B3H 4R2, Canada

**Keywords:** dairy monitoring, social network analysis, precision livestock farming, computer vision, deep learning, cattle identification, behavior detection, animal welfare

## Abstract

This review critically analyzes recent advancements in dairy cow behavior recognition, highlighting novel methodological contributions through the integration of advanced artificial intelligence (AI) techniques such as transformer models and multi-view tracking with social network analysis (SNA). Such integration offers transformative opportunities for improving dairy cattle welfare, but current applications remain limited. We describe the transition from manual, observer-based assessments to automated, scalable methods using convolutional neural networks (CNNs), spatio-temporal models, and attention mechanisms. Although object detection models, including You Only Look Once (YOLO), EfficientDet, and sequence models, such as Bidirectional Long Short-Term Memory (BiLSTM) and Convolutional Long Short-Term Memory (convLSTM), have improved detection and classification, significant challenges remain, including occlusions, annotation bottlenecks, dataset diversity, and limited generalizability. Existing interaction inference methods rely heavily on distance-based approximations (i.e., assuming that proximity implies social interaction), lacking the semantic depth essential for comprehensive SNA. To address this, we propose innovative methodological intersections such as pose-aware SNA frameworks and multi-camera fusion techniques. Moreover, we explicitly discuss ethical challenges and data governance issues, emphasizing data transparency and animal welfare concerns within precision livestock contexts. We clarify how these methodological innovations directly impact practical farming by enhancing monitoring precision, herd management, and welfare outcomes. Ultimately, this synthesis advocates for strategic, empathetic, and ethically responsible precision dairy farming practices, significantly advancing both dairy cow welfare and operational effectiveness.

## Simple Summary

Dairy cows are social animals that form relationships, compete for food, and interact in ways that affect their health and well-being. Traditionally, monitoring these behaviors required humans observing cows directly, which could be stressful for the animals. Now, using artificial intelligence (AI), cameras, and sensors, researchers can precisely track and analyze cows’ movements and social interactions without disturbing them. This review explores how advanced technologies, specifically deep learning and social network analysis, help us understand cow behavior under commercial barn conditions in real time. We explain how tools like object detection, behavior tracking, and facial recognition identify individual cows and monitor interactions. However, current technological systems continue to face challenges in clearly distinguishing between friendly and aggressive behaviors. We also discuss ethical considerations, including potential stress on cows from continuous monitoring and the need to help farmers understand these advanced systems. Overall, this work supports the dairy industry’s move towards smarter, kinder, and less invasive farming practices.

## 1. Introduction

Dairy cows are inherently social animals. They actively participate in both affiliative and agonistic social interactions with other cows within the barn. Affiliative behaviors such as grooming and resting in proximity to others serve to reduce stress while strengthening social bonds. On the other hand, agonistic behaviors such as headbutting and displacement tend to result from competition and the establishment of dominance. Social interactions are important because they influence animal welfare, health, and productivity [1].

Social network analysis (SNA) provides a robust framework to explore and quantify these social behaviors. It offers a computational analysis of interaction maps to examine individual social preferences and group-level cohesion. SNA is becoming more common in the study of dairy cows alongside other research elements, such as computer vision, deep learning, and artificial intelligence (AI). These advancements help with the unobtrusive monitoring of dairy cows, enabling the collection of interaction data and analysis of social behaviors to be conducted on a larger scale [2].

Therefore, understanding cow sociality through SNA methods can support the development of practical welfare-oriented strategies for barn management, in turn, improving cow health, longevity, and productivity. However, most existing AI-driven SNA frameworks prioritize proximity metrics over behavioral intentionality (e.g., distinguishing grooming from forced displacement), limiting their capacity to model true social agency in dairy herds [3]. Considering such constraints and the growing importance of AI in monitoring livestock, a comprehensive synthesis of current approaches is necessary. This review focuses on three main areas: (1) understanding the affiliative, agonistic, and dominance behaviors of dairy cows and the application of SNA as a metric to capture these behaviors; (2) discussing the impact of computer vision, deep learning, and AI technology on monitoring behavior, predicting actions, and constructing social networks; and (3) outlining gaps in the research pertaining to precision livestock farming, specifically those relating to monitoring dairy cows.

### 1.1. Literature Search and Selection Methodology

A systematic literature review was conducted following PRISMA 2020 guidelines [4] to maintain methodological transparency, reproducibility, and academic rigor. The review sought to consolidate the literature on SNA of dairy cows, paying particular attention to studies that integrate computer vision, deep learning, and AI technologies for monitoring and behavioral analysis of cattle.

The primary search was conducted using Web of Science, Scopus, Google Scholar, and ScienceDirect. To enhance discovery and mitigate publication bias, Litmaps was used for citation chaining and cluster mapping to find papers that are semantically related but might not be accessible in traditional search listings. The keywords strategy consisted of combining and permuting terms, including “Social Network Analysis”, “Social Network Analysis of Dairy Cows”, “Precision Livestock Farming”, “Cow Identification”, “Cow Tracking”, “Cattle Keypoint Detection”, “Cow 3D Tracking”, “Proximity Interactions”, “Herd Health Monitoring”, and “Cow Pose Estimation”.

The review focused on studies published from 2019 to 2025 to capture recent advancements like transformer-based architectures, multimodal sensing technologies, and edge inference platforms with real-time capabilities. However, there was a selective addition of studies outside the temporal scope prior to 2019 under well-defined inclusion criteria. These older studies were retained only when they were shown to offer foundational concepts in machine learning applicable to current animal monitoring technologies or provided novel AI-based cattle monitoring technologies that have been substantively validated or extensively referenced in contemporary research. This mitigates temporal bias while still meeting the needs of the review’s theoretical underpinnings.

Eligible studies were those conducted in English, published by a peer-reviewed journal, and focused on monitoring dairy cattle behavior, social interactions, or welfare. The selected studies employed SNA methods to quantify behaviors, including affiliative grooming, agonistic displacements, dominance, proximity-based interactions, and implementation of computer vision or deep learning algorithms or sensor technologies such as Radio Frequency Identification (RFID), ultra-wideband (UWB), and automated milking systems (AMSs) for monitoring cattle. This review analyzed empirical simulations and conceptual frameworks relevant to SNA and AI in livestock systems. The inclusion criteria extended to theoretical sources only when they served a foundational explanatory role, like Alpaydin’s Introduction to Machine Learning [5], which served to explain supervised learning paradigms that are essential for several of the models present in the included research.

Studies were excluded if they were not original research, had no relation to dairy cattle, lacked any application of AI or social network techniques, or failed to include animal science and animal-level behavioral modeling. Additional exclusions were made for papers that lacked methodological clarity or could not be reproduced due to insufficient reporting.

The screening followed a three-stage process. First, a total of 311 records were obtained: 280 from databases, and 31 from manual and Litmaps-aided citation discovery. After removing three duplicates, 308 records remained for title and abstract screening. In this phase, 68 papers were excluded due to a lack of relevance, originality, or not being in English. Two hundred forty papers proceeded to full-text review. During this stage, 112 additional papers were excluded: 41 were on topics unrelated to AI and cattle monitoring, 38 did not focus on animal science, and 33 were deemed methodologically unsound and unfit.

**Figure 1:**
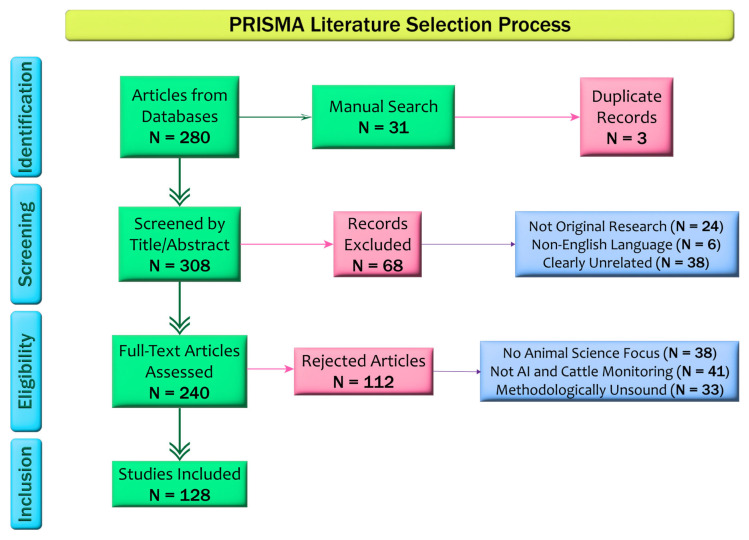
Flowchart illustrating the systematic literature search process following PRISMA guidelines. It outlines the methodical approach taken to select relevant studies on dairy cow behavior, social network analysis, and AI-based monitoring technologies.

The detailed selection process, including the number of records identified, screened, and excluded at each stage, is visually summarized in Figure 1 in accordance with PRISMA 2020 guidelines.

In total, 128 papers were deemed eligible for inclusion. These papers cover a range of subtopics such as behavioral analysis with SNA, identity tracking through object detection models, proximity sensing, and inference of social structure through network metrics. A number of included studies utilized modern architectures such as YOLO, EfficientDet, CNN-LSTM (Convolutional Neural Network–Long Short-Term Memory) hybrids, and attention modules like CBAM (Convolutional Block Attention Module). The quality assessment was performed based on reproducibility and clarity benchmarks that involved data transparency, the availability of source code or algorithms, and the strength of model validation methods (e.g., cross-validation and multi-farm validation).

Several strategies were used to minimize bias. No studies were excluded based on result positivity or statistical outcomes, which, along with the multiple sensing modalities and geographic locations included, ensured that the review was not narrowly tailored to a specific context. Also, citation tracing with Litmaps allowed the inclusion of strong studies that are often ignored to construct a more comprehensive corpus.

This approach provided a well-defined yet comprehensive collection of literature regarding the application of SNA in dairy cow monitoring systems in relation to AI and practical farming applications.

### 1.2. Review Scope and Structure

This review aims to summarize the recent findings and advancements that have been made in the field of SNA of dairy cows with particular emphasis on computer vision, deep learning, neural networks, and AI. It starts by explaining social behaviors in dairy cows and the development of sociality. It then explains the application of SNA for the quantification of social interactions, followed by detailing the technological improvements in dairy cattle monitoring systems. Further sections provide a detailed account of deep learning, object recognition, identity tracking, and interaction inference. Finally, the review examines the ongoing issues in classifying behaviors and synthesizes the identified research gaps alongside future scopes of work.

## 2. Social Network Analysis

Understanding social interactions in dairy cattle starts from looking at the basic social behaviors that define their social lives. This section discusses some SNA results retrieved from analyzing affiliative and agonistic behaviors of dairy cows, the development of social roles, and network-level patterns such as dominance structures, stability, and the impacts of regrouping. Affiliative behaviors in dairy cows are the friendly, stress-buffering interactions that knit the herd together. They include allogrooming—one cow licking another’s head or neck—along with choosing to rest beside favored companions, engaging in midmorning bouts of social licking often tied to feeding, and maintaining long-term “affinity pairs” with specific partners. Collectively, these cooperative acts strengthen bonds, promote calm, and sustain group cohesion and overall welfare. Agonistic behaviors, in contrast, are the competitive exchanges that shape the dominance hierarchy and regulate access to limited resources. They range from headbutting and physical displacements to threat or avoidance gestures and bursts of aggression around feed, water, or resting areas. Although such interactions can elevate stress, they are integral to establishing social order and ensuring predictable, structured use of shared barn space. The review also explains how AI, especially vision-based systems, improves the monitoring and modeling of these behaviors in real time and connects social behaviors to welfare and productivity outcomes.

### 2.1. Grooming Relations and Affiliative Behavior

Grooming among cows is a type of interaction that is systematic, structured, and non-random, and grooming behaviors are often asymmetrical [6]. However, reciprocal grooming is commonly observed in cow herds—suggesting a tendency for cows to groom those who had previously groomed them. It is also interesting to note that cows who heavily invested in grooming others were less likely to groom themselves, suggesting that high social spenders may bear some costs [7].

Stable preferential partnerships, often referred to as “affinity pairs”, have been identified as a key feature of social organization at the herd level, with cows maintaining contact with specific partners [8]. In addition, social licking behavior appears to be widespread and commonly observable in most cows and tends to reach its greatest intensity around mid-morning, often accompanied by feeding. Furthermore, a weak but positive association between proximity and social licking indicates that spatial closeness may be a good indicator of social bonds. Hence, using spatial closeness as an interaction metric is feasible for observing social cohesion within the herd [9]. Familiarity is important since familiar cows had a higher likelihood of grooming each other than unfamiliar ones [10], and social grouping influenced their access to resources and general behavioral patterns [11].

### 2.2. Development of Sociability During Weaning

The foundation for the development of adult cattle social behavior is laid very early in their lives, during their pre-weaning and weaning phases as calves. Calves formed stronger bonds with familiar peers; however, these associations lacked long-term stability [12]. Weaning has also been linked to increased social centralization, with some calves possessing relatively strong social roles that spanned several weeks [13].

Heifers raised socially have been shown to exhibit more expansive and cohesive networks compared to those raised in isolation. This illustrates that early social exposure enables strong social integration post-weaning [14]. Also, cows that were born close in time to each other, or were otherwise related, were more likely to form long-lasting social ties [15].

### 2.3. Impact of Grooming and Affiliative Bonds

Affiliative interactions in cattle extend beyond social comfort, influencing a number of more complex effects in cows’ behavior. For instance, postpartum cows placed into unfamiliar groups demonstrated lower lying times and dyadic synchrony—both of which are considered markers of social stress—which underline the buffering impact of stable social companionship [16]. Likewise, affiliative bonds can also mediate dominance effects. Subordinate individuals have been observed to efficiently access food due to tolerant-dominant relationships. This can be defined as an interaction where dominant cows allow subordinate partners preferential access to resources, effectively assisting them.

### 2.4. Dominance and Hierarchy Structures 

Most researchers agree that the dominance hierarchy formed among dairy cows is often non-linear and context-dependent. Factors such as environmental conditions, hunger, reproductive status, and personality traits tend to influence resource access, often outweighing the rank of domination [17]. Additionally, heavier and male calves were more central in the networks, suggesting that physical traits contribute to increased social value within the group [13]. Some researchers employ various dominance scoring techniques, but they all seem to face difficulty in establishing consistent rankings due to clear differences in measurement methods and observational contexts [17]. Current SNA metrics, while descriptively rich, lack validated links to productivity biomarkers—a critical gap for translational precision livestock farming. This illustrates the need for future studies to combine structural network metrics such as centrality or dominance metrics with measurable indicators of welfare, including but not limited to, milk production, lameness, or lying time.

### 2.5. Influence of Parity, Age, and Health

Age, parity, and health status subtly but meaningfully shape dairy cow sociability. Older animals groom their herd mates more often and both give and receive extra licking, while pregnant cows are groomed more than others; yet these preferences are only loosely tied to dominance rank [7]. Patterns of sociability also diverge with experience: multiparous cows form stronger social bonds and generally yield more milk than first calvers, whereas lame [18,19] or otherwise compromised cows—calves and adults—tend to slip toward the edge of the social network, showing lower centrality and fewer strong associations even when they remain behaviorally active [1].

Because social contact is so sensitive to discomfort, an abrupt drop in affiliative engagement often heralds lameness [18] or other illness days before overt clinical signs appear. Typical early cues include fewer grooming bouts, a drift from central to peripheral network positions [12,20], and longer, less fragmented lying bouts—behaviors that reflect pain avoidance, energy conservation, and a cortisol-mediated stress response well before gait changes become obvious.

Detecting these subtle withdrawals has clear management value. Automated vision or sensor systems that track interaction frequency, network position, feeder visits, or queuing order can flag at-risk animals early, allowing prompt treatment and reducing welfare and production losses. Such tools are especially useful for primiparous cows, animals in early lactation, and high-ranking individuals, all of whom show the sharpest behavioral deviations when health falters.

### 2.6. Individual and Spatial Sociability Patterns

Social behavior differs on an individual basis. Individual differences in social behavior remain consistent over time across most individuals [6,8]. The stable behavioral patterns exhibited by individual cows over time indicate underlying sociability traits [18]. Unsurprisingly, it has been proven that individual positions in a social network, such as centrality, are more stable over time than group-level dynamics [2]. In addition to individual traits, spatial placement is essential to understanding herd dynamics. Cows exhibit greater individual differences in resting areas over feeding areas, indicating the influence of comfort and personal space preferences on social expression [8,18]. Additionally, the location within the barn has been linked to differences in the interaction patterns, underscoring spatial influence on sociability patterns [15].

### 2.7. Network Stability

Social networks among cows are structured and often remain stable over time; they are rather structured and orderly [7]. Affinity relationships within herds of cattle tend to remain stable over extended periods [21]. Measures such as degree and centrality show some repeatability, indicating consistency in individual social roles over shorter periods, hence reinforcing the presence of herd-level social structure and a stable social network [13,15].

### 2.8. Consequences of Regrouping

Regrouping disrupts already existing social systems. The addition of new cows to existing social networks weakened them for at least two weeks [8]. Even the resident–resident ties began to diminish, weakening network strength. However, the underlying cause of this destabilization remains unclear. Longitudinal studies are needed to determine if post-regrouping network fragmentation reflects transient stress or permanent social memory impairment in dairy cows. Similarly, the separation of affinity pairs led to increased variability in milk yield by three-fold, underscoring the extent of impacts of social relationships on productivity [21]. Unfamiliar cows possessed lower centrality, often remaining on the periphery of social structures even days after introduction, while familiar cows offered little interaction to newcomers, indicating passive rejection [22].

### 2.9. Agonistic vs. Affiliative Interactions

Agonistic and affiliative networks are distinct and, as such, largely uncorrelated. These relationships remain relatively stable over time in the herd [6]. The grooming networks were observed to be sparse and stable, whereas displacement networks show greater volatility over time. Interestingly, the cows tend to display significantly higher rates of both affiliative (3× more licking) and agonistic (1.3× more displacements) behaviors with their preferred affinity pair, suggesting emotionally charged bonds and frequent encounters for competition and support [1]. Familiarity appeared to have an impact on affiliative behaviors, but agonistic actions were largely unaffected, which suggests that competition for resources might be more uniform as opposed to preference for affinity pairs [10].

The prediction of cow social roles became possible with the rise of computational modeling. Separable Temporal Exponential Random Graph Models (STERGMs) have demonstrated moderate predictive power (r = 0.22–0.49) to estimate centrality using structural network features, with accuracy notably enhanced by using triangle-based features in place of dyadic metrics. This underscores the growing possibility of short-term behavioral forecasting using graph-based models. As discussed above, SNA illuminates rather sophisticated and subtle social interactions of dairy cows that can be studied and interpreted through quantification. From early-life bonding, affiliative grooming, and the disruptive influence of regrouping, spatial positioning, and hierarchy, bovine behavior is intricate yet remarkably individualistic. Individual traits such as centrality, association strength, and closeness as social traits remain stable across varying times and contexts, providing reliable behavioral identifiers, or “fingerprints,” for each animal [6,8].

### 2.10. Bridging AI and Animal Ethology

Until recently, artificial intelligence and dairy cows were seldom mentioned in the same breath; now, rapid technological advances have brought them together and propelled significant progress across the dairy industry. Now, with the use of AI such as computer vision and deep learning models, monitoring dairy farms has become more efficient and accurate. Instead of needing field staff to constantly walk about the farm with clipboards collecting data, cows can now be monitored through unobtrusive cameras that have been placed around the barn; feeding time, licking time, and even idling can all be tracked, giving remarkable insights into the health of the herd as a whole [23].

A contemporary computerized dashboard enables instant access to numerous performance indicators, such as grazing time, feeding, and standing duration, which are very useful KPIs (Key Performance Indicators) to determine comfort and productivity. Withstanding time in alleys being unproductive, lying time directly represents the feed-to-milk conversion ratio, which significantly impacts profitability and welfare [16]. With this technology, the assessment of welfare has shifted from periodic manual checks, which were very labor-intensive, to something performed on a daily basis in real time, enabling automated evaluations that would have been impractical before [24].

In addition, the cow comfort index (CCI), which was only occasionally applied in academic work, has turned into a measurable standard on commercial farms, where it is tracked daily by AI sensors and cameras [25]. The CCI—the proportion of cows in contact with a stall that are actually lying—summarizes stall suitability in a single welfare metric, with well-run herds expected to score ≥ 85 %. When the CCI is overlaid on a stall-sharing (lying adjacent) network, pens that fall below this threshold typically map onto subnetworks where low-rank cows have reduced degree centrality or are displaced to peripheral nodes, exposing how physical design and social hierarchy jointly limit resting access. Sister metrics—the Stall Standing Index (<15 %), Stall Use Index (>75 % in pens stocked ≥130 %), and Rumination Index (≈50–60 %)—add behavioral layers that can be encoded as edge weights or node attributes, enabling social network analysis (SNA) to distinguish environmental from social drivers of discomfort; all indices should be sampled at peak rest motivation (≈2 h before and 1 h after milking) to synchronize welfare snapshots with the interaction graph.

What the automated milking systems and cameras provide for cattle is often misunderstood as an unwanted intrusion of privacy and the natural setting of the animals. Along with reducing the manual labor needed on the farm, these modern systems give supervisors and operators the ability to intervene less often but more strategically. Also, modernized barns enable cows to behave freely and naturally, and they have the flexibility to decide when they want to rest, eat, or be milked, which bolsters welfare and overall productivity. However, this technological shift also raises important ethical considerations [26]. The pervasive deployment of vision systems in barns necessitates explicit discussion of farmer–cow data consent frameworks and algorithmic transparency to avoid “digital paternalism” in livestock management.

**Figure 2:**
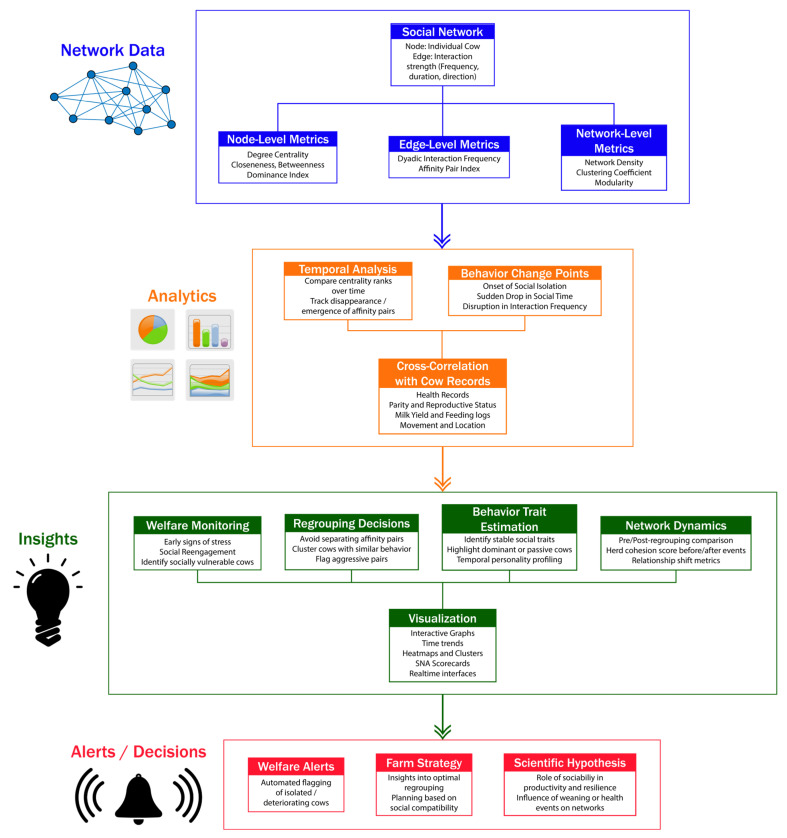
An illustration of the workflow used to construct and interpret dairy cow social networks from collected data. It shows the step-by-step transformation from raw sensor and visual data into actionable insights for herd management and animal welfare.

These visual information streams are mapped by SNA into interpretable social metrics. Indicators like degree centrality, betweenness, or association strength, as listed in Table 1, quantify the level of connectivity and interaction each cow has with the other members of the group, and how central it is to the cohesion of the herd. These are not ungrounded academic abstractions—they constitute practical measures of stress, social isolation, or declining health [18]. Figure 2 provides a visual representation of how social network metrics are processed into actionable insights, management decisions, and welfare alerts.

As technology advances, so does intelligence in the interpretation of camera footage. Modern computer vision systems can now recognize lying, feeding, and even affiliative behaviors such as grooming—all of which integrate seamlessly into SNA frameworks that aid in daily management. To be precise, SNA has transformed from merely a research tool into a real-time welfare system monitoring the health and well-being of animals. It bridges biology to technology to help answer the fundamental question in dairy science: What makes a cow happy? Because, as experts put it, a happier cow is a healthier cow.

To transform social interactions into actionable insights, a key first step is constructing a functional social network for the observed cattle group. Within this framework, social networks constitute graph-structured representations where each individual cow is a node, while social weight interactions between them are denoted as edges. Although directed edges theoretically offer richer relational interplay, most studies today employ undirected networks due to the practical difficulties of determining interaction direction in uncontrolled real-world dairy settings.

## 3. Cattle Monitoring Systems: Enabling Precision Livestock Farming

The ability to evaluate livestock welfare, behavior, and productivity at scale and automatically is provided by automated cattle monitoring systems, which form a part of precision livestock farming (PLF). Wearable and vision-based systems capable of tracking a multitude of behaviors, including feeding, lying, locomotion, and social interactions, have become more widespread because of technological advancements [28]. These systems mark a drastic movement from observation based on manual methods to analytics based on rich data collected in real-time.

### 3.1. From Manual Observation to Semi-Automation

**Figure 3:**
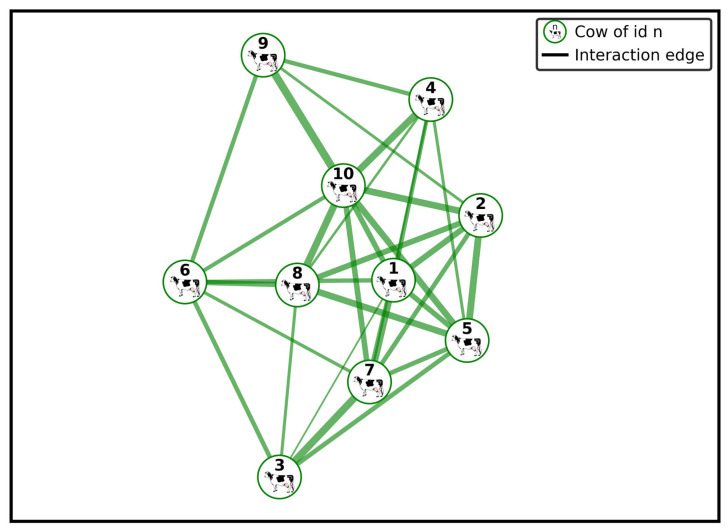
Example of an undirected social network graph depicting interactions among dairy cows. Each node represents an individual cow, and thicker lines indicate stronger or more frequent social interactions, providing insights into herd social dynamics.

In the past, behavioral data collection in cattle studies involved a lot of fieldwork, requiring lots of time and personnel trained to observe the subjects. Scan sampling methods were employed by several studies, where a group of trained human observers note licking and agonistic behaviors in a cow herd with a fixed time interval (e.g., every six minutes), noting their spatial proximity (e.g., within 4 m) [1,7]. This approach is usually conducted over extended periods, such as six weeks. Figure 3 provides a visual example of a social network graph derived from observed interactions among cows within a herd.

Nevertheless, such protocols that are labor-intensive in nature pose issues regarding accuracy and scaling. This is the reason semi-automated systems came into existence. Video analysis software has since allowed trained observers to annotate social interactions in terms of the instigator, receiver, and location, which improved data collection in terms of reliability and efficiency [29]. However, human error is a factor not to be neglected in such methods.

### 3.2. Rise of Smart Farms and Automated Data Acquisition

The cattle monitoring industry has changed very quickly because of the need for real-time, automatic, and situationally aware systems. Advances in animal detection, identification, and behavior classification now allow cattle monitoring under ever-changing, dynamic, and uncontrolled farm environments [30]. In this regard, camera traps, drones, and RGB (Red–Green–Blue)/thermal imaging, along with RFID and GPSs (Global Positioning Systems), have made it possible to acquire behavior-rich datasets from across farms and other settings.

Sort gates demonstrate the level of automation that has been achieved in barn logistics. These gates identified cows through RFID and assisted in their movement for milking, feeding, or health checks. Sort gate passage logs have been used to estimate movement-based affinity pair analysis, which revealed hidden social relationships captured through logistical data [21].

As a passive data source, sort gate logs hold significant promise for welfare-based monitoring. Variations in gate log patterns can indicate changes in social bonding, stress, or illness. While not granularly precise, sort gate data is a valuable, non-invasive tool for tracking individual behavior and well-being within herd systems.

### 3.3. Sensor-Based Monitoring and Network Inference

Technologies that utilize sensors such as GPSs, ultra-wideband RTLSs (Real-Time Locating Systems), accelerometers, and pedometers have become critical for continuous high-resolution positional tracking. Using radio collar tags for triangulation has enabled performing SNA at the herd level, even in populations exceeding 150 individuals [8]. Weighted neck collars equipped with accelerometers allow for precise spatial coordinates estimation [18]. Ultra-wideband RTLS tags that can monitor cow positions at a frequency of 1 Hz have also been employed by some studies for continuous real-time spatial localization of individuals [2,15].

Proximity-based metrics are widely accepted as social interactions, especially within the realm of sensor-based SNA [21]. Although interaction types cannot be defined due to a lack of proximity, cows being close to one another indicates that there is likely an affinity bond forming [1]. Also note that agonistic behaviors of affinity pairs are likely due to locational resource competition due to resource scarcity, which is why proximity is often regarded as an important social metric. Wearable sensors come with numerous advantages; however, data drift, calibration, and external noise slow the sensor operations down, affecting its long-term reliability in outdoor conditions. Additionally, while RTLS tags excel in low-light or crowded barn environments, their inability to distinguish between affiliative behaviors, such as licking, and agonistic ones, like headbutting, renders them inferior to pose-aware vision systems in behaviorally complex contexts. This behavioral ambiguity has driven the development of vision-based systems that offer richer contextual interpretation of social interactions.

**Figure 4:**
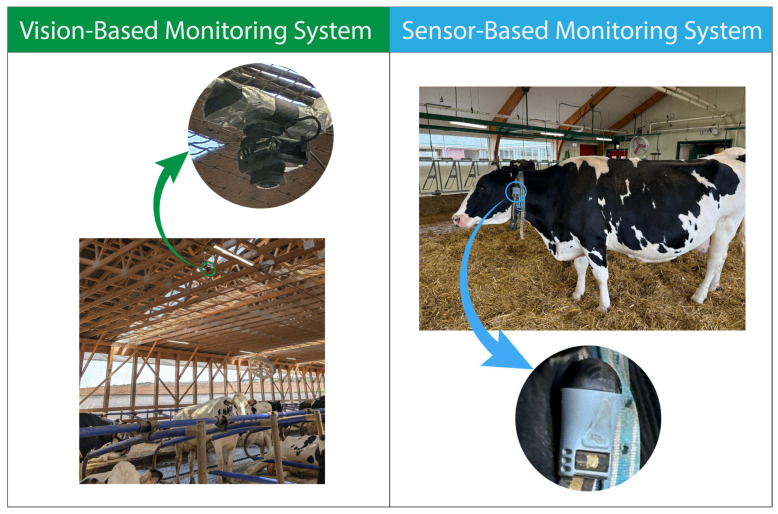
Visual comparison of typical equipment setups in dairy barns contrasting vision-based monitoring systems (using cameras) and sensor-based systems (using wearable sensors), highlighting the differences in complexity, invasiveness, and setup requirements.

Figure 4 visually contrasts these two monitoring paradigms, illustrating how equipment design and data acquisition modes differ in terms of intrusiveness and practical setup requirements within the barn environment.

### 3.4. Advancing Non-Contact and Vision-Based Monitoring

Recent innovations have led to the development of non-invasive, camera-based methods for cattle monitoring. These systems allow for greater insight into behavioral studies without physical contact [31]. This reduces handling stress and facilitates long-term monitoring. One such system integrates computer vision with a weigh scale to estimate milk production and assess udder attributes, achieving 94% accuracy for teat estimation using 3D imaging and R^2^ = 0.92 for yield estimation, showcasing efficiency and scalability in performance monitoring [32]. Similarly, Fuentes et al. [33] developed the first contactless physiological system based on vital signs estimation using RGB and thermal imaging for heart rate and respiration counting. Their system correctly estimated milk yield and composition (fat% and protein%) with a correlation of R = 0.96, highlighting the utility of ANNs (Artificial Neural Networks) as a viable alternative to invasive methods, especially in uncontrolled real-world farm settings.

### 3.5. Computer Vision and Deep Learning for Behavior Detection

With recent advances in deep learning, models like YOLOv5, YOLOv7, and Faster R-CNN (Region-based Convolutional Neural Network) are now available for real-time cattle detection and tracking [30]. These systems outperform traditional detection tools in the identification of interaction behaviors, behavioral deviations, and spatial distribution [34]. Real-world applications of vision systems are hindered by problems like occlusion, lighting conditions, and even the varying breeds of animals. To add to this, the absence of labeled datasets is troublesome for supervised learning approaches [28]. For behavioral inference, pose estimation, mounting, and grooming detection have been implemented using 3D CNNs and LSTMs [35]. However, many of these systems are focused on precise data collection, which makes them prone to errors in uncontrolled and unfamiliar barn conditions.

Table 2 provides a comparative overview of the various sensors, imaging systems, and data acquisition methods used for cattle monitoring, highlighting their respective data types, behavioral functions, strengths, and limitations.

### 3.6. Sensor Fusion and Systemic Challenges

**Figure 5:**
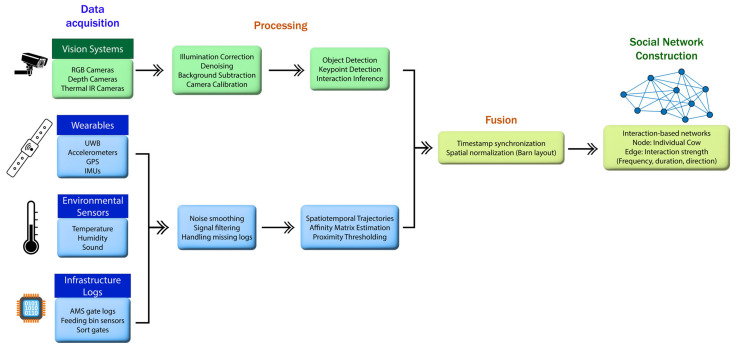
A flowchart representing a multimodal, integrated cattle monitoring system combining visual data, sensor data, and advanced analytics, highlighting how different data streams merge into a unified decision-making tool for enhancing dairy cow welfare and productivity.

The integration of vision, audio, and even wearable sensors into a single framework enhances behavioral inference—these systems are known as multimodality fusion systems [50]. More sensors that enable the recording of cattle behavior must be integrated into wearable devices. Figure 5 visually illustrates the process of integrating multimodal data streams into a unified monitoring framework for constructing a social network.

Even though the employment of tracking algorithms such as DeepSORT in combination with Kalman filters has advanced tracking robustness in high-density cattle areas, merging multi-sensors is still in its early development stages, and field-ready deployments are limited [51]. Additionally, the processing of some data from sensors (such as interpolation, smoothing, and filtering) is much easier than computer vision, which requires a lot of processing to be performed (like image preprocessing, object detection, identification, and tracking). This sets the computational challenge of having efficient and scalable pipelines capable of real-time operation within large farms on-site.

### 3.7. Data Annotation, Quality, and Reproducibility

The dataset’s annotation availability and quality pose a significant bottleneck in model development. Very little research data is available for benchmarking due to the possibility of human errors in manual annotation [30]. The SURABHI (Self-Training Using Rectified Annotations-Based Hard Instances) framework, which uses self-training and label rectification to correct annotation inconsistencies using spatial logic combined with confidence thresholds, provides a viable solution to this issue [52]. This model demonstrated an 8.5% increase in keypoint detection accuracy, which proves that temporal self-correction and attention-based filtering can enhance label robustness in complex frames. AI and sensors are now being used to fully automate the monitoring of cattle, gradually shifting from outdated manual and semi-automated systems. Although vision-based systems and sensor-based systems each have their own unique advantages, their integration, along with better data infrastructure and better annotation, holds significant promise for constructing sophisticated, intelligent, and welfare-oriented farm management systems.

Now that the foundational network behaviors and sociability patterns are mapped and established, it is necessary to explore the computational backbone driving these systems. The next section investigates self-organized neural networks that observe, interpret, predict, and quantify cow behaviors, positions, and interactions at a fundamental level vital for the formation of social networks.

## 4. Deep Learning Algorithms for Computer Vision Tasks

The application of deep learning to cattle monitoring represents a major shift from manual behavioral observation. Deep learning (DL) has shown significant promise in cattle detection, posture recognition, and social interaction analysis, particularly using convolutional and recurrent neural architectures. However, implementing deep learning poses significant challenges due to barn environments, which are obstructed with complex lighting and unstructured movement combined with confined spaces, causing occlusions and limiting the scope of scalable solutions.

### 4.1. Convolutional Neural Networks (CNNs)

For image-based cattle data, CNNs are the most commonly utilized deep learning models. CNNs process data with a grid-like structure, such as images. They use convolutional layers, which apply filters to input data to detect patterns like edges and textures. Tasks that have spatial detection patterns, such as detection, posture classification, and ID recognition, hinge on CNNs.

CNNs have shown mixed and inconsistent performance in cattle monitoring tasks, particularly in uncontrolled barn environments. An Inception-V3 CNN model pretrained on ImageNet, when used on cows’ rear-view video frames to identify them, attained a low accuracy of 57% [53]. This, alongside a few other studies, has highlighted that most baseline CNN pipelines are unusable under dynamic barn conditions with occlusions, dirt, and lighting variations due to their focus on static features and inability to incorporate the temporal context [36]. Though CNNs are effective in detecting static postures (lying down, sitting, and standing), they fall short in recognition of transitions or multiple overlapping behaviors, further fortifying that CNNs are effective in spatial computation but are naive for temporal problems [35].

To overcome the limitations of static CNN pipelines, augmenting temporal models and alternative SVM (Support Vector Machine)-based classifiers have been recommended to improve performance [53,54,55]. High-dimensional feature vectors (e.g., 2048 dimensions) extracted from the CNN pooling layers allow for a feature representation that consists of high-dimensional, information-rich embeddings. While this improves feature representation, it increases the computational cost and may constrain real-time applications. Efforts to lower the computational overhead include modifications to Mask R-CNN architectures, enabling precise back segmentation even under occlusions [56]. At the same time, two-stage models such as Faster R-CNN achieved higher detection accuracy. However, they pose difficulties for use in real-time applications due to their trade-offs in speed and scalability [57,58].

The extensive use of CNNs in livestock applications is corroborated by [59,60,61,62], who reported high accuracies in animal detection and ID classification with CNN-based systems, utilizing YOLOv8 and VGG16 (Visual Geometry Group). Still, pure CNN systems tend to focus on visually distinct features and struggle with tracking social behaviors as they are poor in identifying subtle behavioral cues and temporal continuity, as mentioned earlier.

### 4.2. Spatio-Temporal Modeling and Attention Mechanisms

Perplexingly, CNNs process each frame independently, neglecting the temporal behavior dynamics. This deficiency is addressed by LSTM and BiLSTM networks, which establish dependencies from one frame to the next. They extend beyond the static spatial feature extraction by learning how spatial patterns evolve over time. For instance, incorporating LSTM layers to process a series of CNN features utilizing 20-frame sequences improved identification accuracy from 57% to 91%, highlighting the importance of temporal modeling [53]. Further validating this approach, a CNN-BiLSTM architecture deployed in real-world barn settings achieved greater than 93% accuracy despite heavy occlusions [63]. However, high video frame rate processing of this model leads to enhanced motion detail but poses a worrying issue for constrained edge devices regarding bandwidth due to limited scalability.

BiLSTM networks analyze sequences in both forward and backward temporal directions. BiLSTM models have shown a clear advantage in identity and behavior classification because of their ability to capture subtle motion cues, such as gait and coat pattern shifts. Capable of analyzing spatio-temporal sequences in both forward and backward directions, BiLSTM models have outperformed both CNN-only and Unidirectional LSTM baselines, highlighting the effectiveness of bidirectional sequence modeling [54]. Subsequent studies reported similar findings where BiLSTM exhibited enhanced behavior classification in cluttered environments, especially in integration with spatial CNN features [56,61,64]. To address the problems posed by noisy frames and partial occlusion, attention mechanisms were introduced. An attention mechanism embedding after BiLSTM allows the model to focus on clear, unambiguous identity frames, notably improving the accuracy of the baseline BiLSTM model [64]. Notably, short clips, even as brief as one to two seconds, were efficient, showcasing the data efficiency that attention mechanisms provide. Expanding on sequence analysis, ConvLSTM architectures were employed to preserve spatial structures during temporal modeling. This system was capable of detecting 15 hierarchical behaviors from multiple farms, illustrating strong scalability and robustness in uncontrolled environments [65].

On the whole, these investigations propose that spatio-temporal attentional models are the best for precision livestock farming behavioral analysis concerning livestock detection—though they make the system computationally more expensive, increase latency, and hence, are not well suited for real-time deployment unless optimized.

### 4.3. Transfer Learning and Pretraining

To enhance training efficiency and reduce data dependency, numerous researchers utilize transfer learning from models that are pretrained using large-scale datasets such as ImageNet. A significant improvement was reported on the performance of Inception–V3 after fine-tuning it on rear-view cow images [52,53]. Nevertheless, these pretrained models tend to underperform because of domain mismatch: urban-trained models cannot identify farm-specific features such as tail movement and muddy texture [56,61]. An increase in the performance of YOLOv7 was observed by augmenting it with barn-specific images for retraining, showing how crucial domain adaptation is [30]. However, pretraining also poses challenges—specifically the possibility of overfitting to unnatural augmentations that do not accurately represent real barn variability. As such, although transfer learning can be helpful in accelerating the development of a model, it cannot replace the thorough farm data collection and additional model training needed to fully optimize performance.

### 4.4. YOLO Frameworks for Livestock Applications

YOLO-based architectures are often preferred due to their real-time detection capabilities, enabling on-farm deployments. Their performance, however, often suffers under occlusion, lighting variation, and animal overlap unless specially tailored.

Custom anchor calibration and augmentation strategies applied to YOLOv5/v7 have led to improved detection performance in cluttered contexts [30]. However, pretrained YOLO models from urban or clear farm scenes were unable to accurately localize cattle in occluded contexts, highlighting the need for domain-specific fine-tuning [64]. Addressing this, an attention-enhanced variant, YOLOv8-CBAM, added a Convolutional Block Attention Module, which emphasizes important features. This architecture reached mAP@0.5 96.8% (Mean Average Precision) while precision stood at 95.2%, proving superior in heavily cluttered real-world scenarios as compared to Mask R-CNN and YOLOv5 [29].

Other studies focused on the new variants of Yolo, such as YOLOv5x, YOLOv4, and YOLOv8, all believing in the accuracy–speed ratio offered [65,66,67,68] Even YOLOv7, however, required attention enhancements (such as Coordinate Attention and ACmix (Attention Convolution Mix)) to process dense, object-rich datasets like VisDrone [69].

Although versatile, the YOLO models maintain a competitive edge only with an extensive amount of anchor and attention modular tuning. Moreover, unless combined with a robust tracking system, they heavily struggle with identity switch issues, which need to be addressed for social network tracking. The tracking system, unless it is lightweight, would hinder the real-time capability of YOLO. In practice, deployment feasibility also hinges on energy efficiency: YOLOv8-CBAM’s 40 W power draw per camera significantly limits its scalability in solar-powered or low-resource barns, especially when compared to EfficientDet’s 12 W baseline [70,71].

### 4.5. Capabilities, Challenges, and Future Directions

Neural network models have demonstrated strong effectiveness in the following areas:

- Detection of static postures with CNNs [35,50];
- Behavior recognition with LSTM/BiLSTM and ConvLSTM [53,61,63];
- Real-time detection with YOLO and EfficientDet [72,73];
- Multimodal fusion of RGB, thermal, and spatial data [29,33,74];
- Attention-based frame selection to reduce noise [64,66].

End-to-end models such as EfficientDet preprocess streams of video and, true to their name, achieve real-time inference with fewer FLOPs (Floating Point Operations)—paving the way for edge device and mobile GPU (Graphics Processing Unit) deployment [58,59,66]. The new self-training framework, SURABHI [52], strengthens pose estimation by improving annotations produced by machines, automating an important step in low-annotation data situations. Nonetheless, the development and deployment of efficient neural networks for real-time, real-world use has its own set of challenges.

#### 4.5.1. Key Limitations

Some key bottlenecks regarding neural networks include the following:

- The absence of public datasets greatly limits reproducibility [50,62,75];
- Farm-specific retraining is needed to improve breed, lighting, and occlusion generalization [35,76];
- Real-time detection with YOLO and EfficientDet [72,73];
- The cost of computation for attention-augmented or multi-camera systems still limits deployment in real-time [61,72,77];
- Their applicability for complex spatial behaviors is limited, as many models are tested using single-view data [52,53,54].

#### 4.5.2. Recommendations

The immediate focus regarding the neural network’s use in monitoring dairy cattle should emphasize the following:

(1) Build and share open, multi-farm benchmark sets [33,35,62].
(2) Adopt semi-supervised pipelines with automatic label rectification (e.g., SURABHI) to minimize manual annotation time [52].
(3) Fuse complementary modalities (RGB, thermal, and depth) to harden models against lighting, occlusion, layout, and breed variation [28,51,61].
(4) Design edge-optimized networks that marry compound scaling with lightweight attention blocks for real-time, low-power deployment [53,57,59].

#### 4.5.3. Emerging Directions

The capabilities of precision livestock farming have been enhanced with new automated systems for behavioral understanding, automated tracking, and scalable health diagnostics, benefiting from the innovations in deep learning. But as shown by Arulprakash et al. [58], no single architecture has been proven to satisfy generalizability across multiple domains and problems, such as speed, robustness, and scalability, simultaneously in the context of dairy cow monitoring. Table 3 sheds light on the deep learning architectures employed in recent studies, highlighting how they are tailored to specific tasks in dairy cow monitoring along with their reported performance, advantages, and limitations.

Advancements in deep learning models will stem from innovation in architecture combined with improved infrastructure, including better datasets, smart annotations, and multimodal sensing. Moreover, in practical scenarios, the system’s backbone determines model performance, and dataset quality dramatically compounds the impact. Deep learning models used for dairy welfare and management will gradually shift from analytical tools to components of real-time autonomous AI decision-maker systems, enabling rapid response to monitoring and analytical challenges.

## 5. Object Detection in Cattle Monitoring

**Figure 6:**
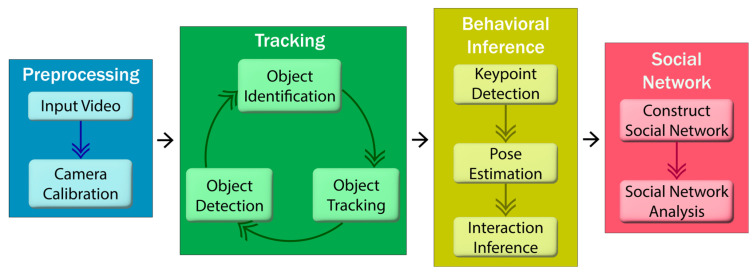
A detailed pipeline overview illustrating the end-to-end process of cattle monitoring using computer vision. This pipeline covers object detection, cow identification, tracking, pose estimation, behavior inference, and subsequent analysis for herd management.

The analysis of cattle social behavior is sequential and hierarchical in nature. It starts with a video capture and ends with an interpretation of interactions among the cattle. Figure 6 shows, in broad terms, the cattle monitoring steps within precision livestock farming.

Although the complete pipeline is defined as the best way to tackle the problem, some of the studies might choose only a smaller subset, such as omitting explicit identification, keypoint detection, or pose estimation based on the data they have, or the goals defined for their problem. Object detection is the first and most foundational step in this pipeline. In this regard, it is essential to explore applied computer vision through the lens of object detection for cattle in barn environments facing occlusion, lighting changes, and movement.

### 5.1. Object Detection: From Static Identification to Context-Aware Sensing

Accurate and robust object detection systems are not only crucial for identifying the presence of cattle but are also fundamental enablers of ID, tracking, pose estimation, and behavior inference. It is desirable for the cattle detection to perform well across a variety of visual distortions, including occlusions, low contrast (e.g., black cattle against a dark background), and irregular movements. The evolution of object detection architectures from early CNN-based models to more accurate, transformer-enhanced hybrid systems has also seen increased complexity.

#### 5.1.1. Early CNN-Based Detection and Two-Stage Architectures

Cattle monitoring initially relied on traditional object detection techniques using CNN-based models along with two-stage detectors such as Faster R-CNN and SSD (Single-Shot Detector). These models claimed to achieve reasonable baseline accuracy with simple and controlled scenarios. A comparative analysis of YOLOv3, ResNet, VGG16, and R-CNN in barn settings demonstrated strong detection accuracy but noted significant limitations regarding real-time inference, occlusion handling, and farm-wide applicability and generalization [35]. Building on this, an enhanced tail detection model built by incorporating Inception-v4 and DenseNet block SSDs, achieved high detection performance (precision: 96.97%, recall: 99.47%, IoU: 89.63%). This model bested YOLOv3 and R-CNN’s accuracy and speed while working with over 8000 annotated images [74]. However, tail-based systems still fall short on occlusion-sensitiveness, severely limiting their application for behavior tracking when animals are densely packed or in motion. Complementing these approaches, CV models such as YOLOv4 have been employed to detect the general presence of cows and classify basic behavioral patterns such as lying, feeding, and walking, providing early indications that computer vision could replace traditional sensor-based systems for continuous cattle monitoring [75].

Around the same time, Andrew et al. [77] tested RetinaNet, another two-stage model, for cow detection. It achieved a mAP of 97.7%, with ID accuracies reaching 94%. RetinaNet did outclass YOLOv3 in classification accuracy but was slower—showing the speed versus accuracy compromise of two-stage detectors.

#### 5.1.2. The Rise of Real-Time Detection: YOLO and Efficiency

Single-stage detectors, particularly variants of YOLO, were adopted to tackle practical considerations around real-time, low-latency inference [83]. Among these, YOLOv5 models provided the best balance of accuracy and speed, with YOLOv7 surpassing detections even more but at a higher cost of computation [76]. Enhancements to YOLOv7 by incorporating attention mechanisms such as Coordinate Attention (CA), ACmix, and SPPCSPC (Spatial Pyramid Pooling–Cross-Stage Partial Connection) increased the model’s mAP by 3–5% while maintaining a real-time frame rate of 30–40 FPS. However, this added computational burden creates difficulties for edge deployment on farms with limited resources or no dedicated GPUs [69].

Comparative evaluations have confirmed that YOLOv5 outperformed Faster R-CNN and DETR (Detection Transformer) in terms of inference time and accuracy, thus further validating YOLO as a real-time cattle detector [68]. Building on this, incorporating attention-based mechanisms, such as CBAM, into YOLOv8 significantly improved its detection performance, outperforming Mask R-CNN and YOLOv5 in highly cluttered scenes [29]. However, the energy efficiency and hardware adaptations needed for optimized energy savings would have to be addressed before any practical use of the system.

A rotated bounding box method for detecting cow heads and torsos instead of traditional edge-aligned rectangular bounding boxes increased the orientation and pose detection capabilities of the detector [84]. Additionally, spatial clustering facilitated more accurate orientation identification. Utilizing the rotated bounding boxes, the use of watchdog mechanisms to prune irrelevant frames showcased a novel method for mitigating computation waste—an essential system design for battery-powered edge devices. Regardless, the influence of lighting and shadow artifacts significantly impacted performance, and thus, the generalizability is limited. Yet, this method provides an important extension of moving from presence-based detection towards pose-informed spatial modeling.

EfficientDet has emerged as a scalable and lightweight alternative to traditional detectors. Leveraging compound scaling with a BiFPN (Bidirectional Feature Pyramid Network), it achieves higher runtime efficiency in FLOPs than YOLOv3 and RetinaNet, making it suitable for mobile and embedded systems [72]. Its effectiveness in human detection propelled its use beyond the scope of reliance to non-human domains, including farm animals. Nonetheless, the accuracy it needs to perform under the specific visual constraints of a barn remains largely untested; hence, it needs dedicated benchmarking against livestock datasets.

While 2D object detection continues to dominate the field of monitoring, efforts toward 3D cattle detection are still at primitive stages. A comparative evaluation of PETRv2 (Position Embedding Transformation) and TransFusion-L, both 3D object detectors, with Faster R-CNN has highlighted their pose awareness and depth perception [51]. However, their exceedingly high resource demand and structured environment dependence render them unsuitable for large-scale deployment in dairy barns.

A common limitation among object detection studies is the use of homogeneous datasets, as they tend to overstate precision while underestimating the variability of real-world conditions [85]. To address this, recent studies strongly encourage cross-domain testing, especially the transition from pristine datasets to unstructured barn settings [57,58]. In addition to broader generalization, hybrid pipelines that combine different detection strategies (e.g., 2D YOLO + 3D PETR + attention modules) have been proposed to improve robustness across breeds, illumination, camera angles, and views. An elementary multi-stage pipeline consisting of YOLOv5x, VGG16x, and SVM/Random Forest classifiers demonstrated moderate success but revealed that this system was highly sensitive to unknown cattle and suffered from ID drift during occlusions [67]. This is a fundamental problem for social network inference that requires reliable identity continuity.

### 5.2. Object Detection as a Foundation for Integrated Behavioral Monitoring

Although object detection forms the computational core of cattle monitoring systems, it is clear from the recent literature that detection alone is far from being sufficient for enabling intelligent behavioral or socially aware models. Object detectors declare the presence of “who is here” but, in the absence of tracking, continuity, pose semantics, and social interactions remain highly opaque. Wang et al. [69] addresses this drawback by incorporating YOLOv7 detection modules with tracking and behavior recognition systems, thus deepening the understanding of cattle motion and interactions. Building further on this, adding a keypoint detection layer immediately after YOLOv8, which is followed by pose estimation and behavior analysis, marks an extension of object detection to behavior cognition [29].

Tracking systems should maintain the cattle’s identity through occlusion and reappearance scenarios across frames. A single-object tracking framework that dynamically combines detection and tracking, where the detector is used to reset identity after a tracking failure, offers a practical solution to this challenge [86]. Building on this approach, instance-level re-ID (re-identification) has been emphasized to ensure continuity in groups, supporting more accurate tracking for performing SNA [87]. Unsurprisingly, the focus on integrated and automated systems is a common thread across different works. The integration of tracking, detection, and pose estimation toward a single predictive engine, which performs appearance modeling and trajectory tracing in parallel, is a commonly supported theme across recent studies [88]. Similarly, hybrid object detection pipelines that combine object detection with scene understanding, pose interpretation, and multi-object tracking have also been proposed to enrich system-level intelligence by making it spatial and subject-aware [57,58]. From a behavioral perspective, the integration of video-based identification systems with open-set re-ID frameworks has proven effective for continuous behavior tracking of both familiar and unfamiliar subjects [72,88]. Recent approaches have explored this pipeline further, linking identity tracking with pose estimation and behavior classification, thereby enabling end-to-end system inference from visual presence to social interactions.

Even efficiently scalable detectors such as EfficientDet can be extended to pose estimation and multi-object tracking, which implies a modular framework for deployment in real-world scenarios [72]. Furthermore, the fusion of identification with activity recognition—linking who the cow is and what the cow is doing—is fundamental for the automated construction of social networks [52,53]. In sum, the literature strongly supports the view that object detection should not be treated as an isolated task but rather as a first layer and a starting point of a more complex system for automated reasoning, which encompasses the integration with identity persistence, motion continuity, keypoint extraction, and labeling behaviors.

## 6. Tracking and Identity Integration Based on Prediction

Cattle often disappear behind obstacles, move through crowded barns, and interact closely with one another, resulting in occlusions. A combination of tracking and detection is necessary in order to achieve continuity across space and time [89]. Thus, detection must evolve into systems that can predict, interpolate, and re-identify animals in sophisticated scenarios [90]. As such, the integration of models based on prediction tracking has surfaced as a focal solution for maintaining behavioral interpretation and long-term identity preservation. 

Incorporating pretrained detectors into regression-based trackers with correlation layers achieved stable bounding box updates over time [87]. This model’s performance improved by ~5% mAP, strongly supporting the value of learned temporal features over simple sequential detection.

In a more structurally sophisticated framework, a Graph Neural Network (GNN) was incorporated to unify detection and multi-object tracking [66]. This system, which was trained in an end-to-end fashion with both classification and contrastive loss, showed an improvement in IDF1 and HOTA scores, meaning identity association and consistency were better. Noteworthily, the GNN’s message-passing paradigm, which captures object relationships across time robustly, provides a key advantage for this model in interaction-rich barn settings. Notably, Tassinari et al. [83] came up with a YOLOv4-based displacement tracker. While it offers a functional lower bound, its lack of predictive mechanisms highlights the problem with naive frame-to-frame tracking, especially in action-dense settings.

To conclude, these models help illustrate that moving from detection to predicting tracking is not simply a matter of performance improvement but rather a shift in paradigm—a fundamental approach and a conceptual necessity. A key shortcoming of systems relying solely on detection is the absence of behavioral modeling in longitudinal approaches, particularly in cluttered or occluded farm environments. However, after considering the latter and as demonstrated by recent models, there is a great necessity and feasibility for integrated detection-tracking pipelines [86].

## 7. Object Tracking in Cattle Monitoring

Tracking an object is particularly important in collecting data for SNA since it allows accurate identification of cow movements based on timestamps throughout the period of observation. Maintaining a consistent identity for each cow over the course of time is important for detecting proximities, affiliative bonds, and even dominance structures. Advances in visual tracking recently experienced a shift from rule-based trackers and sensor fusion approaches to end-to-end trained architectures capable of continuous and real-time monitoring.

### 7.1. Vision-Based Tracking Systems for SNA

Most traditional cattle tracking approaches have relied on sensor–vision fusion systems rather than on standalone vision-based solutions. For example, Ren et al. [91] used UWB tags integrated with computer vision for cow localization, while interaction detection at feeding points was performed through a camera. This approach well served its purpose, but the infrastructure-based nature of its implementation restricts scalability.

On the other hand, Ozella et al. [92] removed the sensors: Object detection was performed at top-down views with EfficientDet, and cow identity was maintained through Euclidean tracking. Importantly, lost track re-identification was performed through trajectory synchronization with milking parlor exits. This illustrates the potential of vision-only systems for automated long-term monitoring of large herds (240 cows in this case) in real time. Though the method relied on predefined infrastructure (milking parlor exit times) for track re-identification, this is something intrinsic to modernized farms and is thus not impractical.

**Figure 7:**
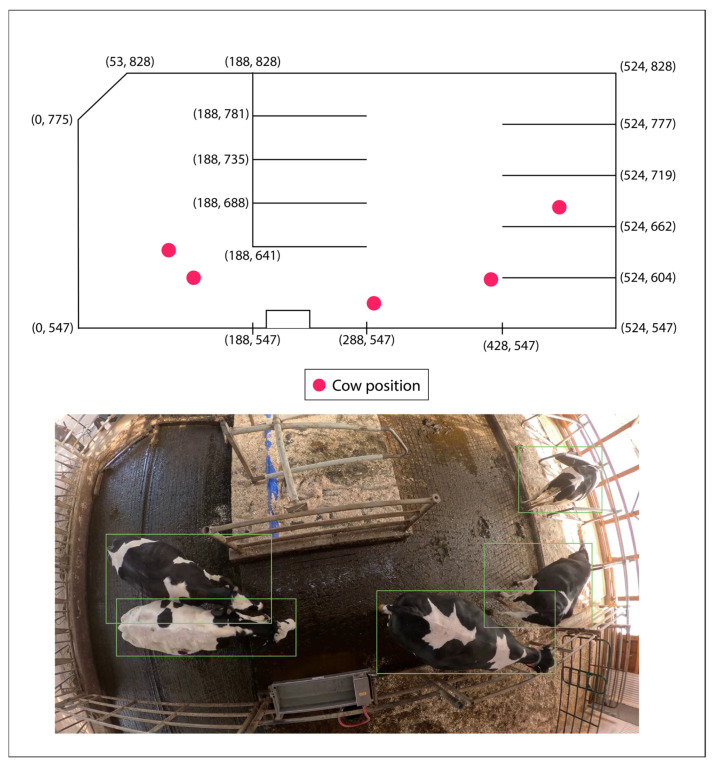
An example barn layout depicting real-time tracking of a small group of cows. The figure shows how cows’ locations and movements within the barn are monitored continuously, aiding in assessing their social interactions and daily activities.

Mar et al. [61] enhanced vision-only systems using a multi-feature tracker that integrated spatial location, appearance features (color and texture), and CNN embeddings. In their pipeline, detection was achieved using YOLOv5, and ID tracking was performed with multi-feature association, gaining 95.6% detection accuracy alongside an estimated tracking accuracy of 90%. Nonetheless, performance suffered greatly due to severe occlusion, underscoring a major challenge of MOT (multi-object tracking) systems: identity fragmentation within cluttered scenes. Figure 7 shows an example of how real-time tracking data of multiple cows is mapped onto the barn layout to visualize cattle positions and movement within the actual farm environment.

### 7.2. Orientation, Keypoints, and Interaction-Aware Tracking

Keypoint-guided tracking has been particularly useful for estimating cow posture, social interactions, and the direction of movement in relation to the herd. Guzhva et al. [84] proposed a rotated bounding box detector based on head, tail, and torso localization. From the probabilistic model and orientation information derived from keypoints, the next frame locations were predicted. Identity tracking was solved using a greedy NMS algorithm. Moreover, their watchdog filtering logic provided a means to cut up to 50% of irrelevant footage while losing only 4% of the meaningful interactions. This shows that intelligent pre-filtering significantly streamlines the entire annotation process without compromising behavioral data.

### 7.3. Deep Affinity Networks and Graph-Based Association

Track-by-detection models frequently suffer from fragmented identities caused by occlusions or missed detections. Sun et al.’s [93] work introduced a design of a Deep Affinity Network (DAN), which learns feature embeddings for detected objects and calculates pairwise affinities for object association across successive frames. The system managed entry, reentry, and exit of objects robustly as well, which enabled its use in the crowded barns scenario with complicated trajectory movements.

Wang et al. [94] built upon this by introducing a graph-based formulation of tracking with min-cost flow optimization. Their innovation, muSSP (Minimum-Update Successive Shortest Path), applied a graph matching approach paired with a minimum-path graph-finding algorithm for bounding box position alignment across two successive frames. By avoiding recalculations in parts of the tracking graph with stable associations, a lot of unnecessary computation is reduced, acting similarly to a high-level graph-based optimization on a DAN. It achieved between 5- and 337-fold acceleration over previous methods while still ensuring optimal association quality. This greatly enhances the real-time computational feasibility of large-scale herd monitoring.

### 7.4. Hybrid Tracking Models: Motion + Detection Fusion

Guzhva et al. [84] designed and used CNN-based tracking with visual markers in top-down views of barns, as they managed to track 23 out of 26 cows for an average of 225 s per session, even in mildly crowded scenes. However, occlusions and visual ambiguity were major limiting factors, particularly in dense scenarios.

Yi et al. [86] further validated the use of CNN-based tracking by creating a hybrid single-object tracker (SOT), which combined a CNN-based correlation filter and optical flow motion compensation. Regular motion was dealt with by the tracker, while a cascade classifier embedded detector dealt with more complex scenarios involving drifts or occlusion events. Their design enhances recovery from tracking failures quite well, and it was tested on standard benchmarks OTB-2013/2015 (Object Tracking Benchmark) and VOT2016 (Visual Object Tracking Challenge).

Complementary to this, real-time detectors like EfficientDet have been adapted for multi-object tracking and pose estimation, which shows how multi-purpose detector frameworks can be redefined to end-to-end tracking systems for track-and-vision integration [72].

### 7.5. End-to-End Architectures for Joint Detection and Tracking

Several modern tracking systems tend to prefer joint tackling of detection and association-based problems, also known as tracking by deep learning [95]. Correspondingly, a Joint Detection and Association Network (JDAN) was developed, which comprised the following: an anchor-free detection head and an association head with an attention-based feature-matching JDAN [96]. Having been trained on MOT16 and MOT17, it outperformed dual-stage baselines on both MOTA and IDF1, offering greater consistency in identity retention and fewer ID switches, even in occluded scenarios. Nonetheless, testing on actual footage from the barn was not performed for this model, marking a gap in practical verification as a key limitation [66].

### 7.6. State-of-the-Art Models: ByteTrackV2 and Beyond

In scenes of high density, which contribute to the failure case scenario of most tracking systems, ByteTrackV2 [51] has emerged as a benchmark. It retains low-confidence detections that are usually discarded by other pipelines, thus enabling continuity of trajectories under occlusion and motion blur. With both 2D and 3D tracking capabilities, it leads the nuScenes and HiEve benchmarks for performance, effectiveness, and accuracy, and even stands as a candidate for real-time deployment in barns. ByteTrackV2′s performance could be enhanced with spatio-temporal modeling by employing transformers.

Also, its active focus on edge devices with low power requirements fits the constraints of on-farm usage. Furthermore, its combination of motion and Kalman filtering provides better continuity for smoother long-term tracking. To resolve identity switches in occluded or crowded barn scenarios, the use of multi-camera fusion with epipolar geometry constraints offers a promising solution [97]. Using the knowledge of barn layout and camera geometry, it is possible to perform preprocessing and consistently triangulate identities across views, which help eliminate fragmentation.

### 7.7. Tracking Benchmarks: A Caution on Generalizability

Some of the tracking systems tested under more academic conditions used datasets designed for humans, such as MOT17, MOT20, and OTB2015. These seemingly robust trackers, which perform well in their specific benchmarks, perform poorly in barn environments due to higher levels of occlusion, irregular trajectories, lower contrast (e.g., black cattle [76]), and non-rigid body deformations [65,94]. As an example, trackers that relatively excelled on MOT17 suffered a 10–15% accuracy drop on the denser MOT20 benchmark [88]. This difference is problematic because it suggests that models trained in a lab are not necessarily robust enough to be freely adapted for use in agriculture.

The development of cattle tracking systems indicates a clear shift towards multimodal, predictive, and identity-aware systems [98]. Systems relying on traditional feature matching have been replaced by more advanced and robust architectures like the DAN [93], JDAN [66], and ByteTrackV2 [51], which perform better under occlusion and crowding. Models still struggle with identity persistence over time, pose-informed tracking, and cross-farm generalization [99,100,101]. Since the social behavior of cattle is highly context-dependent, tracking systems need to further integrate keypoint and pose estimation [84], behavioral prediction [61], multi-camera fusion [64], and end-device optimization for barn deployment in real time.

To conclude, AI and sensors are now being used to fully automate the monitoring of cattle, gradually shifting from outdated manual and semi-automated systems. Although vision-based systems and sensor-based systems each have their own unique advantages, their integration, along with better data infrastructure and better annotation, holds significant promise for constructing sophisticated, intelligent, and welfare-oriented farm management systems.

Now that the foundational network behaviors and sociability patterns are mapped and established, it is necessary to explore the computational backbone driving these systems. The next section investigates self-organized neural networks that observe, interpret, predict, and quantify cow behaviors, positions, and interactions at a fundamental level vital for the formation of social networks.

## 8. Object Identification in Dairy Cows: Approaches, Architectures, and Advances

The seamless and reliable identification of individual cows is fundamental to intelligent monitoring systems for cattle. Identification is critical not only for tracking and behavior analysis but also to link interactions temporally, which is pivotal for social network analysis (SNA). For tracking, verification is a fail-safe step, which ensures the absence of track and id switches. The progress of identification methods in cow monitoring shows an increasing tendency towards multi-view. The advancement of identification methods pertaining to cow monitoring suggests an increasing tendency towards multi-view, multimodal, real-time, and open-set capable architectures. This section reviews the most important changes in cow identification technologies and classifies them according to their technological lineage and imaging techniques.

### 8.1. From AlexNet to Contemporary Pipelines: The CNN Foundation

Modern models for the identification of cows are traced to Alexnet [102], which laid the foundation and revolutionized vision systems as it built a robust multi-layered convolution design and demonstrated a breakthrough performance in the 2012 ImageNet challenge. The architecture of Alexnet, comprising five convolutional and three fully connected layers, set the trend for a new era of deep learning systems in pattern recognition. Other designs that came later, including ZFNet, OverFeat, and VGG, further improved AlexNet by introducing smaller filters, multi-tasking, and sliding window detection [103]. These backbone designs can be regarded as the base from which livestock models were developed.

### 8.2. Identification Pipelines: From Pattern-Based to Re-ID Systems

Pattern-based identification systems in cows depend on cow body coat patterns and require that the patterns are sufficiently unique and temporally invariant. A CNN trained on merely 1000 top-down images of 100 cows (10 different species) achieved 89.95 percent accuracy, thereby validating the claim that body patterns could serve as viable biometric markers [104]. The precision of ResNet-based models can be further enhanced by integrating transformer components (CMT (Convolutional Neural Networks Meet Vision Transformers) modules and Coordinate Attention). The accuracy of CNN-based pipelines has been shown to be notably improved by integrating multi-scale and semantic features [105]. An innovative approach for identification applied a Keypoint R-CNN to identify key anatomical markers, transform triangular body regions into bitmaps, and match using CNNs [106]. This approach was effective and efficient for identification, but it requires highly structured scenarios where geometric consistency across datasets was essential.

### 8.3. Rear View and Lateral Image-Based Identification

Qiao et al. [53] implemented Inception-V3 for CNN feature extraction in video-based identification systems and employed LSTM, BiLSTM for spatio-temporal modeling. This yielded 91% accuracy using 20-frame clips, outperforming standalone CNNs by a wide margin. Similarly, several architectural modifications to ResNet50 increased the rear-view classification accuracy to 98.58% while decreasing model parameters by 24.85×. Various architectural choices, including dilated convolutions, Ghost Modules, and CBAMs, showcased the impact of design decisions on edge deployment feasibility [107].

### 8.4. Top-Down (Dorsal) Views and 3D Identification

Dorsal perspectives are preferable for consistency and reduced occlusion when the animal is walking through the barn passages. Resnet-50 feature embeddings extracted from dorsal view images, coupled with data augmentation, demonstrated improved generalization across varied scenarios [77]. High identification accuracy has also been achieved using SVMs trained on back images, reaching a great accuracy of 98.67% with a group of 48 cows [57]. Further improvement of dorsal detection was achieved through a dual-pipeline approach integrating a custom Mask R-CNN featuring Light_ResNet101 and SE (Squeeze-and-Excitation) blocks, along with features selected using Hu moments and Fisher’s Score. This system (segmentation + SVM classification) achieved over 98% precision and demonstrated real-time operational efficiency with a speed of 1.02 s/frame [108].

The integration of YOLOv8 for detection and VGG for feature extraction, the output of which was fed into an SVM with majority voting across frames, exhibited robust scalability and ease-of-use; however, it is to be noted that the tracking zones were narrow, limiting the area covered for monitoring, and occlusions remained an issue [42]. Building on this further, the CowDepth2023 model integrated both RGB and depth inputs, alongside point cloud data. Their open-set deep metric learning achieved 99.97% using ResNet and 99.36% with PointNet [46]. These are compelling results in support of depth-based identification, especially considering the lighting and occlusion challenges.

### 8.5. Multi-View and Free-View Identification

Multi-view systems assign a special focus on identification problems without a fixed camera perspective. A multi-view re-ID system built with a contrastive and supervised learning-based pipeline attained single image accuracy of over 96% while working with over 100,000 images of 90 cows [90]. Similarly, a free-view recognition model employing YOLOv3 on images taken from variable angles reported 92.2% accuracy in ideal or clean conditions [42]. However, accuracy suffered significantly with overlapping cows, highlighting the need for pose-robust embeddings and occlusion-aware architectures.

### 8.6. Facial Recognition, Keypoints, and 3D Biometrics

The ease of mounting cameras and the uniqueness of faces as biometric attributes have fueled interest in facial identification. Dac et al. [49] applied a full facial ID pipeline deploying YOLOv5 for detection and ResNet101+ArcFace for extracting feature embeddings, and performed cosine similarity search, achieving 93.14% CMC@R1. Further, to improve facial image quality under suboptimal conditions, image denoising and enhancing using autoencoders and GANs (Generative Adversarial Networks) have been applied, with classification performed by Xception and 1D CNN architectures [109]. Complementary approaches have explored metric learning strategies such as Siamese Networks and transfer learning from human face recognition, which have proven particularly effective for sparse datasets [110].

Moreover, 3D keypoints transform identity recognition to pose-aware recognition. Integrating gait and texture information using 3D joint data and grayscale images has proven to significantly improve identification accuracy [111]. At the same time, several dorsal keypoints and the Euclidean distances between them were classified with a Random Forest classification, confirming that body geometry alone can enable accurate identification using infrared imaging, even under varying poses and BCSs (Body Condition Scores) [45].

### 8.7. Identification with Open-Set and No Supervision

Open-set and label-free identification models address real-world herd dynamics. An unsupervised model that combined Cross-Model Contrastive Learning (CMCL) and Adaptive Graph Sampling (AGS) with ResNet encoders, despite the absence of labels, demonstrated resilience to occlusion and variation in lighting [112]. Similarly, an architecture integrating ResNet with a Spatial Transformer (ResSTN) achieved 94.58% open-set accuracy while incorporating four loss functions for embedding robustness [79]. Lightweight models have also shown promise for open-set recognition, a framework built on MobileNetV2 with triplet loss that achieved an impressive 93.8% Top-1 and 98.3% Top-5 accuracy, highlighting the efficiency and real-world viability of compact models for cattle identification.

Table 4 provides a compilation of key features and model architectures used for individual cow identification, along with the imaging perspectives, strengths, and limitations associated with each approach.

### 8.8. Challenges for Cattle Identification

Real-time performance of most models is less than their test accuracies when faced with occlusion, cow pose variations, and overlapping cows [47,60], despite their promising results. Most systems operate under controlled static top-down views and fixed lighting, which seldom exists in commercial environments. The combination of tracking with identification is an unexplored area. Tracking-based ID correction is proposed by only a handful of studies, including [42,46,53], most of whom seem to struggle with even moderately dynamic and dense crowds.

#### 8.8.1. Data Scarcity and Dataset Demands

In addition, there are insufficient empirical datasets available for public access. There is a growing need for larger, annotated, and heterogeneous datasets that reflect the complex, dynamic, and realistic conditions of barns [90,109]. To enable cross-compatible research and create a fair basis for model evaluation, it is recommended to establish benchmark datasets across multiple modalities such as RGB, depth, and IR [47].

#### 8.8.2. Future Directions: Multimodal, Re-ID, and Real-Time Systems

It is not hard to infer that there is a notable push for expansion of identification systems towards integrating multimodal biometric data, including face, body, depth, and IR. Integrating identification with behavioral analysis and health tracking is recommended by multiple studies [46,47,106].

Cross-view and multi-camera ID systems are potential solutions for occlusion issues [64,90]. Open-set and real-time systems need to be optimized and tested extensively for edge device deployment [39,79,105,110].

A clear pathway forward for identification could include the following strategies: self-supervised learning to reduce annotation costs [112]. depth + IR imaging for robust ID under barn noise [45,46], and joint ID-tracking behavior architectures for full SNA integration [42,46,64].

From simple CNN classifiers on rear-view images and coat patterns of cows, cow identification research has advanced to complex hybrid and open-set models trained on depth, keypoints, and top-down trajectories. ID systems today integrate YOLO, ResNet, BiLSTM, transformers, and advanced loss functions for sophisticated fine-grained classification.

However, data and model generalization, occlusion robustness, and real-time deployment still remain as focal bottlenecks. Integrating robust identification models with behavior-aware tracking systems alongside multimodal sensory inputs could be the potential key to scalable PLF and SNA.

To put it aptly, even the coat of a cow is not just a coat anymore, when seen through a modern AI lens, it is a biometric signature with alphanumeric characteristics that needs to be processed, decoded, and identified [104].

## 9. Keypoint Detection and Pose Estimation

Within cattle monitoring systems, keypoint identification resides as a vital intermediary step for Posture-Aware Monitoring. Pose estimation utilizes these keypoints to construct the body configuration of the subject of interest, in this case, cows. Keypoint detection also provides standard methods and abstraction for identification and behavior analysis. By identifying anatomical markers such as shoulders, hips, and spine across frames, these models enable robust biometric identification and temporal tracking under varying orientation and occlusion scenes.

However, uniform texture, deformable body structure, recurrent occlusions, and variation in lighting within barns pose significant challenges in keypoint detection.

### 9.1. Two-Dimensional Keypoint Detection and Structural Geometry Modeling

Identifying dairy cows using infrared imaging and geometric keypoints has shown promising results. By manually annotating several dorsal landmarks, like the hips and tailhead, and calculating pairwise Euclidean distances, a Random Forest Classifier was trained to classify images of individual cows. This geometric representation resulted in high identification accuracy even with occlusion. Especially, the approach was robust to coat color variability and prevailing light conditions, which showcased the importance of structural keypoints [45]. Still, the reliance on manual annotation lacks scalability, particularly in large herds.

To reduce dependence on annotation, a “Locating Key Area” (LKA) model that focuses on key anatomical regions such as the head, torso, and whole body was developed. Though segmentation was performed manually during the training stage, the model demonstrated remarkable generalizability after deployment [114]. Yet, this remains a semi-automated solution that emphasizes the need for a single-step, end-to-end keypoint localization system. Advancing beyond partial automation, a center-frame focused video-based pose model with high structural fidelity [115]. Similarly, template-based approaches, such as skeleton path similarity matching over RGB-D imagery have also been explored [116]. However, these methods are constrained by the lack of 3D modeling and reliance on static templates rather than dynamic tracking.

Eliminating the reliance on predefined templates, Ramesh et al. [106] proposed a zero-shot, keypoint-based instant re-identification pipeline, where triangular dorsal patches formed by the joint keypoints were binarized and stored using a Boolean vector. This method proved useful for identity database expansion without retraining because of its efficiency. Yet, it was too sensitive to slight keypoint inaccuracies, which would cascade through the vector serialization system, greatly diminishing reliability for large, visually homogeneous cohorts.

### 9.2. Three-Dimensional Pose Estimation and Point Cloud-Based Approaches

Realizing the constraints of 2D planar geometry, a number of works progressed toward 3D representations. A Structure-from-Motion (SfM) approach that utilized RGB cow images to estimate cow body dimensions accurately captured wither height and chest girth, dwarfing depth sensor accuracy [117]. This method, however, suffered from direct lighting and reflective surfaces, greatly restricting its applicability in real-life barns.

At the same time, Li et al. [43] used Kinect DK sensors with IR grating to carry out point-cloud-based anatomical slicing. The study applied DBSCAN (Density-Based Spatial Clustering of Applications with Noise) combined with logistic regression for anatomical region segmentation and precise identification of the legs and trunk without the use of deep networks. The system’s generalization to pigs and sheep indicates a robust multi-species applicability. However, real-time scalability is impeded by hardware complexity and computational expense.

### 9.3. Keypoint Detection for Behavior and Identity Fusion

Keypoints have demonstrated dual functionality in both biometric identity and behavior inference [105,111]. Particularly, the variability in postures was implicitly managed in the geometric relationships between landmarks. This is an emerging stream of research that has a strong focus on considering keypoints as not only spatial markers but as behavioral expressions having meaning—this shift is essential for modeling social interactions.

Also, the integration of keypoint and pose modules with the temporal Bi-LSTM networks improved the granularity of behavior recognition [63]. Such pose-temporal integration represents the next advancement towards holistic monitoring systems, where the change in posture over time serves as an indicator for the detection of grooming, lying, or agonistic interactions.

### 9.4. Current Limitations and Research Gaps in Keypoint Detection

Regardless of the considerable work that has been accomplished towards keypoint detection and pose estimation, several limitations persist: Real-time scalability has yet to be fully developed. Current 3D models lack optimization for barn deployment due to their high computational cost [49]. Continued occlusion management remains an important limitation in high-density herds, as body regions are frequently only partially visible. In some form, manual annotation remains the backbone of numerous systems [45,114], which is a hindrance for extensive scaling up. Automated frameworks are, of course a welcome addition, as evidenced by the work of Menezes et al. [45], who implemented a CNN-based keypoint regression network. Further, as reported in [45], real-time implementation was more of a priority, which is why behavior tracking and lightweight integration required more focus.

### 9.5. Necessity for Behavior Inference

In addition to serving as biometric references, identification keypoints and pose estimation are also vital to understanding posture, interactions, and animal health. The landscape of keypoint-based cattle analysis is undergoing rapid transformation, ranging from basic 2D landmark matching to complex 3D reconstruction and zero-shot biometric encoding. However, for these models to be useful in commercial dairy operations, a focus on achieving real-time responsiveness, unsupervised scalability, and integration with downstream behavior inference systems is vital.

In the next sections, the focus shifts to the modeling of interactions and behaviors, elevating keypoints and pose trajectories from anatomic markers to social movement metrics.

## 10. Interaction Inference: Evaluating Contactless Approaches for Social Behavior Mapping

Social networks in dairy cattle are built on social interactions; hence, identifying and understanding these interactions are vital for mapping them. Though manual observation followed by video review (manual observation paired with video analysis, termed post hoc analysis) allows accurate measurement, it is very resource-demanding and lacks scalability. Because of this, more recent research adopts contactless, automatic systems. Most of these systems, however, depend entirely on proximity-based interaction logic, which, while efficient, fails to recognize the incidence of spatial presence as opposed to social interaction.

Ren et al. [91] provide a case of this shift with the hybrid system that integrates RTLS with computer vision for interaction detection in feeding areas. This method allows spatial proximity detection among cows and demonstrates viability for large-scale real-time monitoring; however, it lacks semantic richness. The system cannot assess the nature of interactions (affiliative versus agonistic) and who plays the initiating or receiving role; thus, the system provides undirected social network graphs, which may be considered falsely directed under the right assumptions. Proximity methods such as those in [91] advance the social constructs of networks but limit the network’s integrity, especially in cases when the behavioral quality of the interactions is very valuable.

A rule-based algorithm for detecting displacement events at feeding bins, utilizing timestamps of entry and exit timestamps to infer agonistic behavior, improved the precision of detected interactions [38]. This method was able to measure dominance and determine social hierarchy through asymmetry in interactions. Still, the method is spatially constrained to the external vicinity of feeding bins. Its extrapolative potential to other contexts within the barn or behavioral scope remains unexamined, indicating limitations in behavioral and environmental scope.

A significant improvement is noted in a study by Ozella et al. [92], in which a vision-based system with YOLOv5 and top-down video was implemented. Their system successfully classifies interactions as head-to-head (presumably affiliative) and head-to-body (presumably agonistic). Most importantly, it employs temporal smoothing and ID tracking, making it less sensitive to frame detection noise. On the other hand, the use of distance heuristics as rules for interaction classification lacks behavioral depth. The system works, but not for the fine detail needed for interpretation of complex actions such as grooming, mounting, and subtle displacements. In addition, the robustness of the model across different barn layouts, lighting conditions, and herd composition variability has not been investigated. The requirement of pose-aware representations with anatomical keypoints and joints in motion is recognized but lacks practical implementation in the interaction classification stage. This is quite a critical gap because interaction type tends to highly depend on particular body positions and motion patterns. Bounding volumes and proximity measures on their own are insufficient for classifying interactions.

In sum, while recent studies such as [38,91,92] attempt to automate interaction inference, demonstrating significant progress, they still lack deep semantic or spatial reasoning or are computationally fragile. Systems purely relying on spatial proximity or pre-defined threshold heuristics risk oversimplifying social complexity, particularly in crowded barns. Integrating keypoint detection, pose estimation, and temporal context represents the next step for directionally sensitive behavior-specific interaction mapping and constructing socially valid, rich networks for complex analytical social systems. Table 5 provides a compilation of methods used in recent studies to infer social interactions among dairy cows, highlighting their operational principles, strengths, and limitations across both manual and automated approaches.

To truly distinguish active social engagement from passive spatial co-location, future systems must integrate gaze estimation and ear and head pose tracking to identify intentional interactions instead of relying solely on proximity thresholds. Such refinements would enable systems to interpret not merely whether animals are located in each other’s vicinity but how they are attending to each other, enabling a better classification of behaviors like affiliative, agonistic, or avoidant interactions.

## 11. Behavior Analysis

The analysis of dairy cattle behavior is critical in enabling automated systems for welfare evaluation, productivity optimization, and early-stage disease detection. While traditional methods used wearable sensors like accelerometers and IMUs, more recent studies are leaning toward vision-based deep learning frameworks because of their high scalability, non-intrusive nature, and ability to capture behavioral subtleties. Unfortunately, there is still some work to be carried out, especially regarding occlusion, pose variability, and generalization across farms.

### 11.1. From Sensor-Based Tracking to Vision Models

Early behavior models focused on the use of IMU and accelerometer data, where LSTM frameworks outperformed CNNs in capturing temporal behavioral patterns such as lying and walking [118]. Multimodal integration, combining accelerometer, gyroscope, and magnetometer data, enabled recognition of complex actions with low latencies through structured signal fusion [37]. However, like most systems, they are limited in scope due to high hardware maintenance demands and limited scalability for large-scale farm deployment.

On the contrary, behavior classification using video footage is becoming popular due to its non-intrusive methods. The recognition of dynamic behavior transitions (e.g., lying to ruminating) is improved with spatio-temporal classifiers like C3D–ConvLSTM, overcoming the limitations of static classifiers [81]. Similarly, object detection frameworks using models such as YOLOv5 demonstrated robust action classification even in crowded and low-light scenarios [120]. While these systems achieve impressive accuracy in controlled environments, the validation of their performance in complex, cluttered, and real-world farm settings remains limited.

### 11.2. Posture-Aware Behavior Recognition

Behavior modeling often starts with posture classification as a proxy for action recognition. Systems utilizing CNN-based architectures like ResNet-50 and MobileNetV2 have shown high accuracy in recognizing standing and lying postures [121]. However, these models fall short in differentiating actions during transitions because of their inability to capture subtle temporal changes, for instance, from resting to grooming.

Spatially restricted systems, such as those focusing only on feeder zones, have limited and shallow behavioral scope and fail to generalize to barn-wide or multi-camera contexts [56]. Although zone-specific models are capable of achieving local precision, their limited view hinders general applicability to entire herd monitoring.

Hybrid models that incorporate both posture and motion features, particularly using CNN and BiLSTM architectures, have addressed some of these issues. These models improve recognition for action-rich behaviors like walking and grooming by accounting for subtle temporal changes in joint movement [63]. Despite this, most systems still function on anonymized subjects without integrated identity tracking, which is vital for monitoring individual traits and health.

### 11.3. Advancements in Temporal and Multimodal Architecture

Temporal modeling has emerged as one of the most important aspects of behavior classification. LSTM units capable of sequence-based processing are highly accurate and effective in capturing behavior evolution and periodicity compared to spatial-only CNNs [118]. Temporal Segment Networks (TSNs), being capable of incorporating long-term temporal context, further highlight the limitation of CNN-only architectures [122]. Some systems have implemented pattern correction mechanisms, which stabilize predictions over time and, through recursive learning, enable accurate recognition of over 90 behavior types [123].

Meanwhile, multimodal techniques have proven to be effective in enhancing recognition fidelity. A classification technique guided by image representations of IMU signals is effective in preserving the spatial interdependencies between sensor axes, thus enhancing classification accuracy across overlapping behaviors [37]. However, most of these systems are still under-tested in commercial farm environments, with little emphasis on cross-barn validation or environmental variance [124].

Monitoring psychological factors has also emerged as a behavior proxy. For instance, phase-based magnification combined with optical flow has been successful in capturing subtle signals such as respiration [125]. Yet, such methods require the use of controlled lighting conditions and high-resolution data, making them unsuitable for unsupervised farm deployment.

### 11.4. Towards Practical and Reproducible Behavior Analysis Systems

Although significant progress has been recently made with behavior inference, several key issues are still unsolved:

- Behavioral Depth: Most systems are centered around basic activities, such as standing, lying, and walking, and tend to ignore complex behaviors like aggression, stress indicators, or reproductive cues [126];
- Contextual Inference: Only a handful of models link behaviors to environmental or social context. Integration with pose estimation or attention-based modeling could improve behavior localization and interpretability at a higher level [81];
- Scalability and Real-Time Application: Very few systems focus on and are optimized for deployment on edge devices in large herds or pasture settings [37];
- Lack of Identity Integration: Tracking individual identity is often ignored during behavior prediction, reducing the value of these data streams for personalized monitoring and welfare analytics [43,62];
- Limited Reproducibility: Many studies do not document recording conditions and lack standardized evaluation protocols, greatly limiting reproducibility and adaptation in practical situations [124].

To conclude, behavior analysis systems for cattle have advanced from classification models that relied on wearables to multifunctional, contactless, vision-based systems that cover multiple behaviors. However, the majority of today’s models have restrictive boundaries in real-world generalization, system scalability, and behavioral granularity. For complex farming settings, pose-informed modeling, context-aware classification, and multimodal fusion (like vision with sound or thermal) need to be implemented to achieve robust and actionable behavior inference.

## 12. Research Gaps and Future Directions

While substantial advancements have been made in vision-based cattle monitoring and social behavior analysis, several core limitations continue to hinder the methodological consistency, scope of implementation, and feasibility of real-world deployment. The following gaps outline persisting issues that were substantiated by the literature analyzed in this study.

### 12.1. Incomplete Pipelines Without Comparative Evaluation

While several studies feature partial monitoring pipelines, such as detection and identification ([46,80]) or detection and tracking ([76,92]), very few have shown complete integration from object detection to behavioral categorization to social network graph construction. Not many systematic comparisons have been performed where partially implemented pipelines are set against complete ones by filling the missing modules with standardized models and measuring the SNA output dependencies.

Future studies should prioritize developing methodological benchmarks. Systems with partial architectures in past studies should be extended using standardized, modular, and reproducible techniques (e.g., ResNet for re-ID and BiLSTM for behavior), and benchmarked as end-to-end systems. This sandbox approach, however, requires publicly available open code and datasets for the established studies, which are both lacking. This brings us to the next limitation towards the development of a cattle monitoring pipeline.

### 12.2. Dataset Bottlenecks and the Need for Open, Multimodal Benchmarks

A number of high-performing models from the literature are built from proprietary datasets under controlled settings [37,46,104]. Moreover, the behaviors and interactions also lack standardization for labeling, rendering them impossible to compare between different studies. Very few datasets contain multimodality (e.g., RGB + depth + thermal) or necessitate long-duration tracking for temporal stability analysis within a network. Among the publicly available cattle datasets, only a few support the implementation of a robust continuous monitoring pipeline, and none provide sufficient data for a complete end-to-end pipeline [127].

The absence of open multimodal datasets containing various types of barns, their cameras, and labeled social behaviors poses a problem. Without these, the claimed model performance is context-bound, and studies in the dairy field risk stagnation due to a lack of reproducibility.

Future efforts should aim at the comprehensive creation of open, standardized, multimodal datasets annotated with rich and relevant social behaviors. Such initiatives can adopt the approach taken by the autonomous driving domain, where progress has been rapid because researchers and developers use shared publicly benchmarked datasets like KITTI and WaymoOpen, which are openly available, well-annotated, and used as standard benchmarks for evaluating and comparing models.

### 12.3. Interaction Inference Still Relies on Heuristics

Despite some vision-based systems extending beyond raw proximity, most techniques for interaction inference still operate using static spatial thresholds and are bound by proximity [92]. There is little to no motion or posture cue disambiguation for instigator-receiver role determination. Key behavioral actions such as grooming, butting, or displacement are often oversimplified, and the undirected edges are very commonly considered.

**Figure 8:**
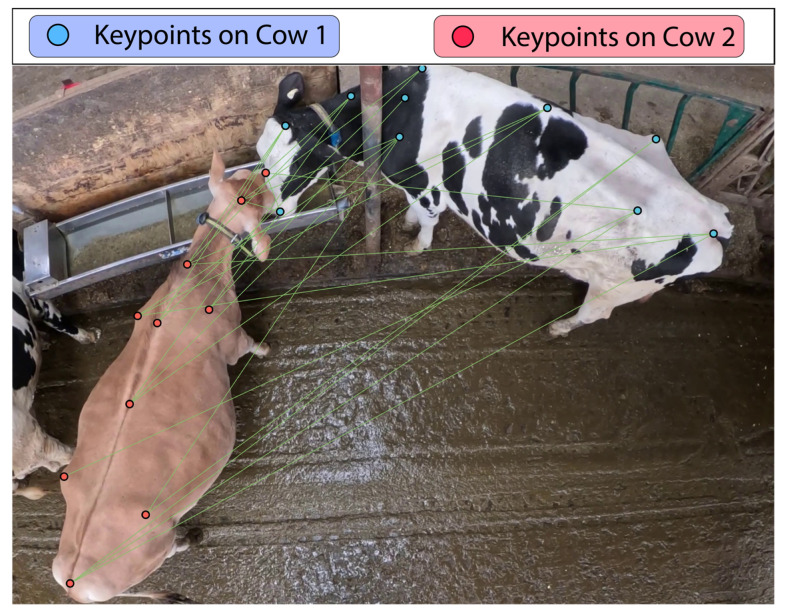
A graphical depiction of the distance between key body points (keypoints) of two cows during a headbutting event. This illustrates how pose-aware tracking methods can distinguish between aggressive interactions and general proximity.

Implementing an interaction classification model based on poses with keypoint trajectory and temporal calibration would fill this gap. Figure 8 provides a visual demonstration of this idea. Labeling interactions at the frame level with real validation will serve to achieve the demands of a holistic interaction detection system, which is currently lacking across most datasets. Analyzing the temporal variation in distance between pairs of keypoints across frames will serve as the key to granular interaction inference.

Future research should prioritize developing standard baseline benchmarked pose-based models trained on annotated temporal sequences of interactions, leveraging spatio-temporal graph networks or 3D CNNs. Designing a standardized annotation protocol could guide the development of datasets containing affiliative and agonistic interactions and cues related to them.

### 12.4. Inability to Generalize in Complex Barn Environments

While many models report high performance under controlled test setups, generalization to free-moving, multi-breed, and occlusion-heavy barns is seldom demonstrated. Studies like [70,80] observed significantly decreased accuracy due to uncontrolled viewpoints or overlapping animals. There is a significant gap in cross-barn, cross-breed, and cross-season generalization of algorithms, models, and datasets.

This requires systematic, multiple, and varied benchmarks compounded with external evaluation—these have been remarkably overlooked in the existing literature. Datasets benchmarked under such varied conditions must be established. Models developed for interaction detection and cattle monitoring in future studies should be pretrained on these datasets to establish a baseline upon which continuous progress can be observed in this field.

To further fortify the solutions, cross-barn and cross-breed benchmark challenges should be complemented by collecting and organizing datasets that are multi-seasonal and multi-location as well. Researchers should focus on designing domain adaptation and transfer learning strategies tailored to livestock monitoring contexts.

### 12.5. Towards Comprehensive Behavioral Taxonomies for Welfare Assessment

Most behavior recognition systems concentrate on 2–3 high-level categories such as feeding, lying, or walking, and very little work has been performed to expand taxonomies, for example, on social behaviors, stress indicators, or reproductive cues [43,56,77]. There are studies that were able to recognize as many as 15 behaviors [65], but there is no established benchmark for behavioral taxonomy hierarchies that are deemed important for health and welfare analytics.

Future work should focus on designing a developed behavior taxonomy that is both layered, structured, and encompasses social and individual actions. The taxonomy can be complemented by using interdisciplinary consensus involving ethologists, veterinarians, and AI researchers. These should be incorporated into open and standard datasets for training and testing universal behavior recognition models.

## 13. Ethical Considerations in Precision Livestock Farming

The increasing integration of AI and automated surveillance systems in dairy barns has created new ethical predicaments regarding data ownership, algorithmic transparency, and the digital rights of both farmers and animals.

As data is one of the most valuable resources in PLF, questions arise regarding who gets to own the data that captures the behavioral and biometric features of dairy cows—the farmers or the third-party monitoring system service providers? Farmers spend capital on sensor systems and data infrastructure, but the processing and insights are often handled by proprietary algorithms from third-party vendors and developers. In the absence of clear frameworks, this can result in unfettered and asymmetric control over access to livestock data by the service providers. The models interpreting the livestock are not usually simple and understandable for the farmers, and in addition, they might not have the proper knowledge and training to access the dairy cow monitoring data on their farm.

Just as troubling are the black box decision-making algorithms of cameras and tracking systems. Modern AI-driven analytics increasingly influence management actions ranging from selective milking to automated health alerts. The absence of explainable AI and transparent model documentation leaves farmers dependent on trusting the opaque systems, risking overdependence on tools they cannot audit and have control over. This situation raises the threat of digital paternalism, where algorithmic control over the barn decision supersedes human oversight without recourse.

In addition, although the concept of animal privacy might seem like an overly humanized perspective, continuous video monitoring and biometric surveillance of individual cows indeed require ethical framing. Although cows cannot consent to being monitored, it is the responsibility of researchers, engineers, and the dairy monitoring system designers to implement systems that would avoid causing stress, behavior distortion, unreasonable intrusion, and respect animal welfare [128].

For these reasons, the pervasive deployment of vision systems in barns necessitates proactive attention to farmer–cow data consent policies and algorithmic transparency so as not to incur “digital paternalism” in livestock management. On a broader scale, AI in agriculture requires an ethics-first approach prioritizing participatory design, equitable data governance, and regular ethical auditing to ensure that PLF technologies serve as a tool of empowerment in the field, rather than instruments to control, invade, and subjugate.

## 14. Conclusions

The integration of advanced artificial intelligence into dairy barns has lifted the curtain on the previously unseen social complexities of cows, challenging conventional views and opening new avenues for compassionate herd management. Traditionally, the social lives of dairy cows were simplified and observed through basic human observation, leaving critical details overlooked. However, the emerging combination of social network analysis (SNA) with cutting-edge AI technologies provides a powerful lens to decode the subtle social cues, affiliations, and hierarchies within dairy herds. The transformative potential of this approach reaches far beyond mere academic curiosity; it stands to significantly enhance animal welfare, farm productivity, and ethical farming practices.

At the heart of this review lies a profound novelty: the explicit integration of sophisticated AI methodologies such as transformer architectures and multi-view tracking into traditional SNA frameworks. These advanced AI techniques offer a significant methodological leap forward, enabling precise tracking and interpretation of individual and group interactions with a depth previously unattainable. Although systems like convolutional neural networks; recurrent architectures, such as BiLSTM; and detection models, including YOLO and EfficientDet, have achieved notable progress in behavior classification, detection, and identification, they still fall short of fully capturing the intricacies of cow interactions. Current methods rely heavily on simple proximity metrics, inadequate for accurately differentiating intentional social interactions like grooming, aggression, or social affiliation from mere passive proximity. Such limitations hinder our ability to accurately interpret herd dynamics and consequently restrict practical applications in farm management.

To directly confront these shortcomings, this review explicitly underscores innovative methodological intersections. Pose-aware frameworks and multi-camera fusion methods represent novel avenues to enhance semantic richness and improve the granularity of interaction interpretation. Pose-aware tracking leverages keypoint detection to recognize specific behaviors and intentional social signals between cows, moving well beyond basic proximity measures. Similarly, multi-camera fusion addresses critical problems like occlusion and lost animal identity, common hurdles in practical barn settings. These methodological innovations significantly strengthen the robustness and accuracy of AI-driven cattle monitoring systems, thus elevating their utility in real-world farming scenarios.

Moreover, this review uniquely emphasizes ethical dimensions within precision livestock farming, raising compelling questions about animal welfare, data governance, and algorithm transparency. Continuous surveillance methods, although technically promising, carry the risk of inducing stress and anxiety among cows, which are sensitive animals capable of complex emotional responses. The adoption of advanced AI technologies must, therefore, be accompanied by a thoughtful ethical framework prioritizing animal well-being and transparent data management. Such ethical considerations not only bolster the credibility of these technological systems but also foster acceptance and trust among farmers and consumers alike. This explicit acknowledgment of ethical responsibility represents an essential novelty and provides a solid foundation for sustainable long-term deployment of AI in dairy farms.

In terms of practical, real-world applications, the implications of integrating advanced AI methods with SNA are substantial and immediate. These methodologies empower farmers with precise, real-time insights into herd health, behavioral changes, and social dynamics, directly translating into informed management decisions. For instance, the ability to detect subtle shifts in social interactions can serve as an early warning sign for stress or illness, enabling timely intervention and reducing the economic impact of health-related disruptions. Additionally, recognizing stable social affiliations and hierarchies informs grouping strategies, mitigating stress during regrouping and optimizing milk production. Hence, these technological advancements hold significant promise not just theoretically, but practically, offering tangible benefits to daily farm operations.

Nevertheless, achieving the full potential of AI-driven cattle monitoring systems depends on addressing several critical challenges. The lack of standardized, openly accessible multimodal datasets with comprehensive behavioral annotations remains a major barrier to reproducibility and generalizability across different farm contexts. Without robust, standardized benchmarks, progress in this field risks becoming fragmented, slowing the advancement of universally applicable AI solutions. Future research must thus prioritize the creation of openly available datasets, comprehensive annotations, and modular validation protocols applicable to real farm environments.

Ultimately, the convergence of AI, animal ethology, and SNA embodies more than a mere technical evolution—it signifies a philosophical transformation in dairy farming practices. This shift calls for viewing dairy cows not merely as producers of milk but as social beings deserving of nuanced understanding and care. Integrating advanced AI methodologies with SNA promotes an empathetic, ethically responsible approach to animal management, profoundly enhancing welfare and productivity. By embracing this compassionate technological ethos, the dairy industry can achieve operational excellence while fostering a deeper respect and understanding of animal life, setting a powerful precedent for future agricultural practices.

## Figures and Tables

**Table 1:** Sociability metrics for evaluating dairy cow social interactions.

Metric	Definition	Behavioral Interpretation	Calculation Method	Data Requirements	Limitations	References
Degree Centrality	Number of direct connections	Measures social popularity; high = frequent interactions	∑ edges per node	Interaction logs	Ignores interaction quality	[2,20,27]
Betweenness	Role as a social bridge	Identifies gatekeepers controlling resource access	Paths passing through node	Network topology	Computationally intensive	[2]
Closeness	Average path length to others	Reflects social integration; low = isolated individuals	1/∑ shortest paths	Full network data	Sensitive to network size	[12]
Eigenvector Centrality	Influence within network	Highlights cows central to cohesive subgroups	Adjacency matrix eigenvectors	Weighted interactions	Favors high-degree nodes	[12,14,15]
Association Strength	Frequency of pairwise interactions	Indicates affinity bonds or avoidance	Interaction count/time	Continuous tracking	Context-dependent	[12]
Reciprocity	Mutual grooming/displacement	Measures social balance; high = reciprocal relationships	Mutual interactions/total	Directed interactions	Fails in dominance hierarchies	[7,14]
Network Density	Proportion of realized connections	Group cohesion; high = tightly knit social structure	Actual edges/possible edges	Complete interaction data	Biased by group size	[10]
Dominance Index	Asymmetry in agonistic interactions	Hierarchy stability; high = clear dominance order	Wins/(wins + losses)	Agonistic event logs	Misses subtle competition	[17]
Clustering Coefficient	Tendency to form triangles	Subgroup formation; high = cliquish behavior	Triples of connected nodes	Local network structure	Less meaningful in small networks	[8,18]
Reachability	Access to others via indirect paths	Social integration; low = marginalized cows	Binary reachability matrix	Full network	Binary simplification	[22]
Synchrony Index	Temporal alignment of behaviors	Social bonding; high = coordinated resting/feeding	Cross-correlation of timelines	High-resolution tracking	Requires timestamped data	[20]
Social Differentiation	Variation in interaction rates	Individual sociability traits; high = diverse social roles	Standard deviation of interactions	Longitudinal data	Sensitive to observation duration	[18]
Edge Persistence	Stability of pairwise ties	Long-term social preferences	Interactions over time windows	Multi-session tracking	Requires repeated measures	[15]
Affinity Pair Score	Strength of preferential partnerships	“Friendship” bonds; high = stable grooming/resting pairs	Dyadic interaction frequency	Individual-level tracking	Environment-dependent	[21]
Isolation Index	Proportion of time alone	Welfare risk; high = social withdrawal	Solo time/total time	Location + interaction data	Confounded by barn layout	[18]

**Table 2:** Data collection methods for cattle monitoring.

Device/Method	Data Type	Explored Functions	Advantages	Limitations	References
Scan Sampling	Interaction Data	Social Network Construction	Highly accurate	Labor-intensive	[1,7]
UWB sensors	Sensor Spatial Data	Tracking	Scalable, accurate, easy to implement	Only distance-based behavior interaction metrics can be inferred	[2,12,36]
IMU sensors (acceleration, gyro, magnetometer)	Spatial Inertial Measurement Units	Tracking, Behavior Analysis	Accurate behavior classification, lightweight	High power consumption, limiting scalable, long-term, on-device usage	[23,37]
AMS Infrastructure Logs 1	Sort Gate Data + Milk Yield	Social Network Construction	Passive data source, reasonably accurate herd-level behavior monitoring	Individual-level behavior monitoring is not feasible	[21]
2D Camera	RGB Images	Detection, Tracking, Identification, Keypoint Detection, Behavior Analysis	Real-time, cost-effective, scalable	Occlusions, lighting variation not handled well	[14,38,39,40,41]
AMS Infrastructure Logs 2	Electronic Feed, Water Bin Visit Time (Entry, Exit)	Social Network Construction	Lightweight, passive data source, reasonable herd-level social network	Social network constructed is limited to the observed region and not generalized well	[42]
Multiple 2D cameras (Multi-view)	Multi-View RGB Images	Detection, Tracking, Identification, Keypoint Detection, Behavior Analysis	Handles occlusions, Id switches well, real-time, cost-effective, scalable	Standardized multi-camera fusion models not available	[43,44]
IR Camera	IR Images	3D Keypoint Detection, Identification	High accuracy, robust to coat patterns	A lot of manual annotation required, limited real-time applicability	[45]
2D Camera + IR	RGB-D Images	Identification, Keypoint Detection	High accuracy	Computationally expensive	[46,47]
Thermal Camera	RGB + Thermal Images	Identification, Keypoint Detection	High accuracy, robust to indistinct coat patterns	Methods for deployment in real farm settings underexplored	[48]
Kinect DK Camera	3D (RGB-D) Images	3D Keypoint Detection	Generalizes well across species	Relies heavily on manual annotation	[49]

**Table 3:** Comparison of deep learning architectures used in dairy cow monitoring.

Model	Application	Performance Metrics	Computational Cost (TFLOPs)	Strength	Limitations	References
YOLOv8-CBAM	Detection	mAP@0.5: 96.8%, 95.2% P	40 W/camera	Occlusion robustness	High energy use	[29,71]
EfficientDet-D4	Detection	mAP@0.5: 94.1%, 12 W	5.6	Edge device optimized	Struggles with small objects	[72]
BiLSTM + Attention	Identification	96.67% accuracy	28	Temporal context modeling	Requires video sequence	[64]
Mask R-CNN	Segmentation/ID	94% IoU, 98.67% ID	22	Precise instance segmentation	Slow for real-time implementation	[57,78]
Vision Transformer	Open-set ID	99.79% CMC@1	45	Scale-invariant features	Needs large datasets	[79]
DeepSORT + YOLOv5	Tracking	MOTA: 82.6%, IDF1: 89.4%	18	Occlusion handling	ID switches in dense groups	[76,80]
ResNet-50 + ArcFace	Facial ID	93.14% CMC@1	8.2	Lightweight embeddings	Frontal view required	[48]
ConvLSTM	Hierarchical behavior	84.4% F1-score	33	Spatio-temporal modeling	Computationally heavy	[81]
ByteTrackV2	Multi-object tracking	HOTA: 68.9%, IDF1: 76.2%	14	Balances speed/accuracy	Struggles with erratic motion	[51]
PointNet++	3D ID	99.36% accuracy	21	Depth-invariant features	Requires RGB-D sensors	[46]
DenseNet-121	Facial ID	97% accuracy	6.7	Feature reuse efficiency	Overfits small datasets	[82]
STERGM	Network prediction	r = 0.49 (centrality)	N/A	Dynamic network modeling	Requires historical data	[2,15]
SURABHI (Self-train)	Pose estimation	+8.5% keypoint accuracy	9.1	Reduces annotation effort	Initial manual labels needed	[52]
Graph Neural Network	Multi-object tracking	89% precision	19	Reduces computational cost	Detection, tracking tradeoff	[66]

**Table 4:** AI-based methods for individual cow identification.

Identification Feature	Model	Camera/View	Strengths	Limitations	References
Coat Pattern	CNN Identification	Top-down body photos	Coat patterns were shown to be viable biometric fingerprints	Sensitive to image quality, pose variation, lighting conditions	[56,104]
Coat Pattern	RetinaNet	Top-view torso images	Efficient one-stage detection, robust to lighting, viewpoint, class imbalance	Low tolerance to occlusion, bounding box threshold, training data quality, limited real-time scalability	[70]
Coat Pattern	FAST + SIFT + FLANN	Side-view images	High accuracy, scalable and efficient for real-time use	Vulnerable to visually similar cows, asymmetric coat patterns, lighting, environment variation	[113]
Coat Pattern	Resnet-18	Multi-top-down body photos	Good performance in confined environments without manual annotation	Not robust to occlusions, varied lighting conditions, texture-invariant herd	[90]
Coat Pattern	YOLOv3	Non-fixed point of view images	Flexible over data sources, multiple angles, and effective for real-time use	Poor performance with occlusions and group images	[60]
Cow Back Pattern	Mask R-CNN + SVM	Top-view images	Accuracy > 80% for behaviors including licking, headbutt	Limited to the feed bunk area in AMS context	[57]
Video-Based ID with Temporal Motion	Inception-V3 + LSTM/BiLSTM (+ Attention)	Rear-view video	Temporal modeling greatly improved ID accuracy vs. single-frame CNN	Accuracy decreases when the cows are static or have minimal movement, which is common in dairy barns	[53,54,64]
Facial ID	Resnet101 + ArcFace	Frontal face images + thermal	Recognizes cattle facial biometrics accurately, akin to human face ID	Affected by lighting/angles	[48]
Muzzle Pattern	YOLOv5+Transformer	Muzzle images	Scalable, one-stage, real-time, robust to partial occlusion	Needs quality images, high training cost, complexity due to transformer, feature loss due to cropping	[68]
Body Anatomical Keypoint Geometry	Random Forest Classifier	Top-down view IR imaging	Robust to similar coat patterns, varying BCS, poses and lighting variation	Heavily reliant on manual annotations, limited real-time capability	[45]
3D Motion + Coat Pattern	RGB Depth Maps + SIFT	Lateral RGB-D videos from both sides	Robust across viewpoints, lighting conditions, text-invariant herds	Requires RGB-D infrastructure and sensitive to occlusion	[111]

**Table 5:** Methods of inferring social interactions among dairy cows.

Method	Definition	Strengths	Limitations	References
Manual observation	Scan sampling method used to observe and record interactions in the herd by human observers	Highly accurate affiliative, agonistic interaction data	Labor-intensive, involves human errors, human limitations in observing the whole area, leading to missed interaction observation	[1,7,9]
Continuous video-based observation	Affiliative, agonistic interactions identified with aid of analysis software	High-resolution behavior identification, with varied interaction types	Labor-intensive, limited scope for automated system integration	[6,10]
UWB RLTS	Proximity-based interaction inference	Suitable for PLF	Cannot identify interaction type	[2,8]
UWB RLTS + accelerometer	Proximity-based interaction inference	Automated and scalable, can detect spatio-temporal patterns	Cannot differentiate between affiliative and agonistic interactions	[18]
Rule-based using video at feed bunk	Detects displacements via feed bunk entry and exit times	Infers dominance hierarchy	Limited to feeding context	[38]
AMS infrastructure record-based analysis	Affinity pairs identified by association in sort gate, milking parlor passing times	Simple, efficient, non-invasive, scalable way to identify affinity pairs	Cannot distinguish interactions, and may conflate dominance, friendship, avoidance	[19,21]
EdgeNeXt + IMU	Behavior classification using acceleration, angular velocity, and magnetometer data from IMU devices	High accuracy (95.85%), includes social licking detection	Licking, neck and leg rubbing were identified, but primarily for skin disease detection	[37]
LSTM + IMU	LSTM-based RNN trained on time-series IMU data to predict behavior	Good accuracy and includes social licking: 80.3%; headbutt: 81.9%	Frequent misclassification of licking and headbutting due to short duration, similar movement, fluctuating IMU patterns	[118]
RLTS tags + LSTM (vision)	Detects interactions at feeders using RLTS proximity and LSTM vision	Accuracy > 80% for behaviors including licking, headbutt	Limited to the feed bunk area in AMS context	[91]
CNN detection	Object detection and proximity threshold-based interaction detection	Non-contact method of interaction inference	Does not differentiate interaction type	[84,119]
Vision-based (YOLO + tracking)	Classifies head-to-head vs. head-to-body interactions	Adds semantic info and temporal smoothing	Uses distance heuristics and misses subtle behavior	[92]

## Data Availability

Not applicable.

## Abbreviations

The following abbreviations are used in this manuscript:

ACmix	Attention Convolution Mix
AGS	Adaptive Graph Sampling
AI	Artificial Intelligence
AMS	Automated Milking System
ANN	Artificial Neural Network
BCS	Body Condition Score
BiFPN	Bidirectional Feature Pyramid Network
BiLSTM	Bidirectional Long Short-Term Memory
CA	Coordinate Attention
CBAM	Convolutional Block Attention Module
CCI	Cow Comfort Index
CMC	Cumulative Matching Characteristic
CMCL	Cross-Model Contrastive Learning
CMT	Convolutional Neural Networks Meet Vision Transformers
CNN	Convolutional Neural Network
ConvLSTM	Convolutional Long Short-Term Memory
DAN	Deep Affinity Network
DBSCAN	Density-Based Spatial Clustering of Applications with Noise
DETR	Detection Transformer
FAST	Features from Accelerated Segment Test
FLANN	Fast Library for Approximate Nearest Neighbors
FLOP	Floating Point Operation
GNN	Graph Neural Network
GPS	Global Positioning System
GPU	Graphics Processing Unit
HOTA	Higher Order Tracking Accuracy
ID	Identification/Identification Score
IDF1	Identification F1 Score
IMU	Inertial Measurement Unit
IR	Infrared
JDAN	Joint Detection and Association Network
KPI	Key Performance Indicator
LKA	Locating Key Area
LSTM	Long Short-Term Memory
mAP	Mean Average Precision
MOT	Multi-Object Tracking
MOTA	Multiple Object Tracking Accuracy
OTB	Object Tracking Benchmark
PBVM	Phase-Based Video Magnification
PETR	Position Embedding Transformation
PLF	Precision Livestock Farming
R-CNN	Region-based Convolutional Neural Network
re-ID	Re-identification
RFID	Radio Frequency Identification
RGB	Red–Green–Blue
RGB-D	Red–Green–Blue + Depth
RTLS	Real-Time Locating System
SfM	Structure-from-Motion
SIFT	Scale-Invariant Feature Transform
SNA	Social Network Analysis
SOT	Single-Object Tracker/Tracking
SPPCSPC	Spatial Pyramid Pooling–Cross-Stage Partial Connection
SSD	Single-Shot Detector
STERGM	Separable Temporal Exponential Random Graph Model
SURABHI	Self-Training Using Rectified Annotations-Based Hard Instances
SVM	Support Vector Machine
TSN	Temporal Segment Network
UWB	Ultra-wide Band
VGG	Visual Geometry Group
VOT	Visual Object Tracking
YOLO	You Only Look Once
